# Diffuse Optical Tomography Using fNIRS Signals Measured from the Skull Surface of the Macaque Monkey

**DOI:** 10.1093/texcom/tgab064

**Published:** 2021-11-10

**Authors:** Ryusuke Hayashi, Okito Yamashita, Toru Yamada, Hiroshi Kawaguchi, Noriyuki Higo

**Affiliations:** Neurorehabilitation Research Group, Human Informatics and Interaction Research Institute, National Institute of Advanced Industrial Science and Technology (AIST), 1-1-1 Umezono, Tsukuba-shi, Ibaraki 305-8568, Japan; Computational Brain Dynamics Team, Center for Advanced Intelligence Project, RIKEN, Nihonbashi 1-chome Mitsui Building, 15th floor, 1-4-1 Nihonbashi, Chuo-ku, Tokyo 103-0027, Japan; Neural Information Analysis Laboratories, Department of Computational Brain Imaging, ATR, 2-2-2 Hikaridai Seika-cho, Sorakugun, Kyoto 619-0288, Japan; Neurorehabilitation Research Group, Human Informatics and Interaction Research Institute, National Institute of Advanced Industrial Science and Technology (AIST), 1-1-1 Umezono, Tsukuba-shi, Ibaraki 305-8568, Japan; Neurorehabilitation Research Group, Human Informatics and Interaction Research Institute, National Institute of Advanced Industrial Science and Technology (AIST), 1-1-1 Umezono, Tsukuba-shi, Ibaraki 305-8568, Japan; Neurorehabilitation Research Group, Human Informatics and Interaction Research Institute, National Institute of Advanced Industrial Science and Technology (AIST), 1-1-1 Umezono, Tsukuba-shi, Ibaraki 305-8568, Japan

**Keywords:** diffuse optical tomography, functional near-infrared spectroscopy, macaque, nonhuman primate neuroimaging

## Abstract

Diffuse optical tomography (DOT), as a functional near-infrared spectroscopy (fNIRS) technique, can estimate three-dimensional (3D) images of the functional hemodynamic response in brain volume from measured optical signals. In this study, we applied DOT algorithms to the fNIRS data recorded from the surface of macaque monkeys’ skulls when the animals performed food retrieval tasks using either the left- or right-hand under head-free conditions. The hemodynamic response images, reconstructed by DOT with a high sampling rate and fine voxel size, demonstrated significant activations at the upper limb regions of the primary motor area in the central sulcus and premotor, and parietal areas contralateral to the hands used in the tasks. The results were also reliable in terms of consistency across different recording dates. Time-series analyses of each brain area revealed preceding activity of premotor area to primary motor area consistent with previous physiological studies. Therefore, the fNIRS–DOT protocol demonstrated in this study provides reliable 3D functional brain images over a period of days under head-free conditions for region-of-interest–based time-series analysis.

## Introduction

Functional near-infrared spectroscopy (fNIRS), a noninvasive neuroimaging technique, optically measures hemodynamic changes coupled with neural activity. This technique estimates changes in oxygenated and deoxygenated hemoglobin concentrations (HbO and HbR, respectively) in cortical blood flow using changes in absorbance of the near-infrared light emitted from the source optode, passing through the head tissues and recorded at the detector optode. The spatial resolution of fNIRS is low (~5–10 mm), and its imaging depth is restricted to ~20–30 mm from the human head surface due to the limitation of light penetration in the diffusive tissue (Haeussinger et al. 2011; Tremblay et al. 2018). However, fNIRS has relatively high temporal resolution in terms of signal recording and tolerance against motion artifacts compared with other noninvasive neuroimaging techniques used for estimating cerebral blood flow dynamics, such as functional magnetic resonance imaging (fMRI) and positron emission tomography (PET). In fMRI and PET measurements, participants’ body and head movements inside the scanner are severely constrained. In contrast, fNIRS enables measuring participants’ brain activities under fewer body movement constraints. The recent development of portable fNIRS systems should render neurological experiments easier and less expensive than other methods and even make it possible to record brain activities under naturally behaving conditions.

Although fNIRS allows participants’ movements during recording, it is necessary to remove artifacts originating from body movements. In cases where the fixation of a holder that attaches the optode to the participant’s head is insufficient, the optode fluctuation alters the optode–scalp gap distance and causes motion artifacts in fNIRS measurements (Umeyama and Yamada 2013). Furthermore, physiological signal contamination other than cortical blood flow changes is a major source of fNIRS artifacts (Takahashi et al. 2011; Yamada et al. 2012; Scholkmann et al. 2014). When fNIRS signals are measured from the scalp surface, the effect of blood flow changes in the scalp tissue is almost equivalent to that in the cortical tissue.

Earlier, we proposed a novel experimental system using nonhuman primates (macaque monkeys) (Yamada et al. 2018). In our experimental system, fNIRS signals were measured from the optodes directly affixed to the skull surface for stable recording from the same positions without the signal contamination from scalp. We reported that the signal sensitivity measured in our system is sufficiently high to capture significant increases in HbO and decreases in HbR at the channels close to the motor-related areas contralateral to the hand movements. Nevertheless, the channel-wise analysis in the previous study was limited to identifying changes in brain activity within anatomically defined individual brain regions. This was because the channel position indicating the largest activation in such topographic measurement does not necessarily indicate the foci in the cortex. This is a common problem encountered in the channel-wise analysis of both monkey and human fNIRS data, so it should be overcome through a new analysis protocol.

Diffuse optical tomography (DOT) (Bluestone et al. 2001) is an imaging method that determines three-dimensional (3D) volumes of light absorption in diffusive media, such as head tissues, by measuring the signals of light propagated in the medium from its surface. Recently, several DOT algorithms, as extensions of multichannel fNIRS, have been proposed to provide 3D imaging of cortical hemodynamic changes with improved spatial resolution and finer signal source separation, especially in the depth direction (Shimokawa et al. 2012; Yamashita et al. 2016; Tremblay et al. 2018). However, the choice of optimal DOT algorithms and parameters depends on the experimental setup, noise level, and other recording conditions. Hence, it is essential to investigate the conditions required for reliable DOT image reconstruction considering the property of the measured experimental data.

In this study, we focused on cerebral DOT and examined how the 3D distribution of hemodynamic response changes can be restored from the fNIRS data in which major artifacts are substantially eliminated. In the Materials and Methods section, we briefly explain our fNIRS experimental system. We then describe the framework of DOT algorithms and the conditions we tested for the comparison study. In the results section, we apply the DOT algorithms to the measured fNIRS data and report the reliability of DOT reconstruction over different recording dates.

The contributions of our research are as follows: 1) we applied DOT algorithms to the fNIRS data recorded from a nonhuman primate for the first time; 2) the optimal DOT algorithms and conditions were examined for the experimental data without signal contamination due to scalp blood flow, in which the elimination of such contamination is a major issue in DOT algorithm studies; 3) we demonstrated that DOT image reconstruction could be stably calculated up to a finer scale close to a submillimeter voxel size (0.6-mm cubic voxel); 4) we observed reliable significant activation at the motor-related areas, including areas in the sulcus, contralateral to the hand movements; and 5) we distinguished the peak latency of hemodynamic response changes in individual brain areas. These findings and results support the validity of our fNIRS–DOT protocol, that is, the combination of DOT and fNIRS measurements from the skull surface, to reliably investigate 3D brain activity under head-free conditions for region-of-interest (ROI)-based time-series analysis.

## Materials and Methods

### Animals and Care/Experimental Protocols

We originally reported the data from this study in the previous papers (Yamada et al. 2018; Kato et al. 2020). Briefly, we used two male Japanese macaque monkeys that were older than 5 years. The protocol of the present study was approved by the Institutional Animal Care and Use Committee of National Institute of Advanced Industrial Science and Technology (AIST) in accordance with the guidelines within the “Guide for the Care and Use of Laboratory animals” (Eighth ed., National Research Council of the National Academies). We described the details of the animal care protocols in the previous paper (Kato et al. 2020).

### Positioning of Optode Sockets Using MR Images

The positions of primary motor and premotor areas were first determined using stereotaxic coordinates from the magnetic resonance (MR) images of the monkey’s brain using a 3.0T MR scanner (Philips Ingenia 3.0T, Philips Healthcare, The Netherlands). We used a T1-weighted turbo field echo sequence (repetition time/echo time, 7.3/3.2 ms; the number of excitations, 2; flip angle, 8°; field of view, 134 × 134 mm; matrix, 224 × 224; slice thickness, 0.6 mm; number of slices, 200) and a T2-weighted turbo spin echo sequence (TR/TE, 1500/283 ms; NEX, 2; fip angle, 90°; field of view, 134 × 134 mm; matrix, 224 × 224; slice thickness, 0.6 mm) to obtain MR images for identifying the anatomical structures of individual animals. Under anesthesia and sterile condition, we incised the scalp around the parietal region, including the motor areas, and fixed optode sockets on the skull surface using a mixture of acrylic resin and titanium oxide, matching the optical scattering property to that of the skull. We described the details of the surgical procedures in the previous papers (Yamada et al. 2018; Kato et al. 2020).

### fNIRS Recording

**Figure 1:**
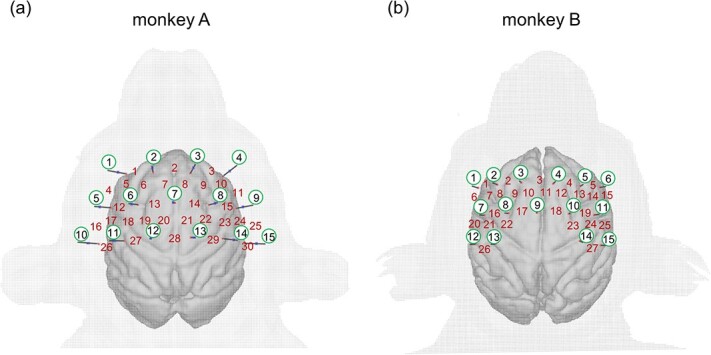
The arrangements of the fNIRS optodes and recording channels for the two monkeys. We selected optode pairs at the nearest neighbor as recording channels covering both hemispheres around the parietal regions, including the motor cortex. Black bars indicate the directions and positions of optodes. The optode numbers are inside the green circles, and the channel numbers are in red letters between the source and detector optodes. (a) The 30-channel arrangement in monkey A. (b) The 27-channel arrangement in monkey B. This figure is adopted and modified from Figure 1 in the paper published by (Kato et al. 2020). Monkeys A and B correspond to monkeys 2 and 1 in the study of (Kato et al. 2020), respectively. Refer to Supplementary Fig. 1 in the Supplemental Material for top, right, and left views of the optode positions.

We recorded fNIRS signals using a triangular bidirectional optode arrangement (Yamada et al. 2018) that covers the parietal regions of the monkey’s head as schematically depicted in Figure 1. The source–detector distance was fixed as 15 mm with a spatial interval among adjacent channels of 7.5 mm. We used the OMM-3000 (Shimadzu Corporation, Japan) to switch between illumination and detection in each optode. We recorded the optical absorbance data at wavelengths of 780, 805, and 830 nm for each channel and used 30 channels (monkey A) or 27 channels (monkey B) for the measurements. The absorbance data bidirectionally measured from the same optode pairs were averaged as one sample with a time interval of 130 ms (sampling rate of 7.69 Hz). The details about our recording system were described in the previous papers (Yamada et al. 2018; Kato et al. 2020).

### Experimental Task

We trained the animals to retrieve a small spherical food pellet (5 mm in diameter) from the Klüver board with a cylindrical well (11 mm in diameter) (Yamada et al. 2018; Kato et al. 2020). Each animal sat in a chair under head-free conditions during the experiments and retrieved the food pellets through a slit approximately every 20 s using each hand alternately. The animals underwent 150 trials (75 left-hand trials and 75 right-hand trials) in each daily session. The onset time of food retrieval movement was detected using a Digital Laser Sensor (LV-11SB with sensor head LV-S72, Keyence, Japan) and recorded concurrently with the fNIRS signals for event-related signal analyses. We recorded the fNIRS data for 4 days from monkey A and for 3 days from monkey B. The total number of left-/right-hand trials was 300 for monkey A and 225 for monkey B.

### fNIRS Data Processing

We converted the raw voltage data into log-ratios using a base-10 logarithm and applied digital low-pass (Butterworth filter of order 7, cutoff 0.7 Hz) and high-pass (Butterworth filter of order 3, cutoff 0.01 Hz) filters. We further removed the mechanical artifacts using a previously proposed method (Umeyama and Yamada 2013) that is based on the fact that changes in absorbance caused by body motion or noise exhibit different spectroscopic properties from those related to tissue hemoglobin absorption. Then, we segmented each run into trial data starting 5 s before the hand movement onset and ending 20 s after the onset of the task. We defined the task onset time as 0 and set the pretask period as [−5 0] s. The baseline for each trial was adjusted to the average amplitude during the pretask period and set as 0. We calculated noise variances from the data during the pretask periods.

### Diffuse Optical Tomography

Because the head tissue is a highly scattering medium, the isotropic light propagation inside the head tissue can be described by the diffusion approximation of the radiative transfer equation (Tremblay et al. 2018). If the absorption changes due to hemoglobin variation are small relative to the absorption in tissues themselves, the Rytov approximation leads to a linear relationship between ***y*** (the log-ratio of the light intensity changes measured from *M* pairs of source and detector probes) and ***x*** (absorption changes in the *N* voxels of the discretized head tissue), as follows (Arridge 1999; Durduran et al. 2010; Shimokawa et al. 2012; Yamashita et al. 2016):

(1) \begin{equation*} \boldsymbol{y}=\mathbf{A}\boldsymbol{x} \end{equation*}

where **A** denotes the sensitivity matrix representing light transmission from sources to detectors across the head tissue.

Reconstructing ***x***, light absorption in a 3D volume, from ***y***, the boundary measurement of optical signals, is inherently an underdetermined inverse problem (Habermehl et al. 2014). Specifically, countless distributions of ***x*** within the volume can explain the same boundary measurement ***y*** when *N* ≫ *M*. Therefore, solving this inverse problem requires a priori information to constrain possible solutions. One approach to solving the ill-posed inverse problem of DOT is introducing a regularization term in a cost function and estimating the optimal solution about absorption inside the head tissue by minimizing the cost function. The solution estimated in this manner explains the boundary measurements while satisfying the a priori constraint described as a regularization term. Another approach is Bayesian modeling that uses probabilistic models of observations and constraints, referred to as the likelihood function and prior distribution, respectively, to calculate the posterior distribution (Guven et al. 2005; Abdelnour et al. 2010). The Bayesian modeling approach we used in this study (Shimokawa et al. 2012; Yamashita et al. 2016) assumes the hierarchical Bayesian model and uses the variational Bayesian method to compute the approximated posterior distribution by solving the maximization problem of the *“free energy”* function.

Studies comparing different DOT algorithms have demonstrated the advantage of the Bayesian modeling algorithm over the simple regularization method in terms of less blurred signal source estimation (Shimokawa et al. 2012; Yamashita et al. 2016; Tremblay et al. 2018). This is because the Bayesian modeling algorithm can implement more refined a priori information about hemodynamic responses in the brain, such as the sparseness constraint in which brain activity in response to a certain task is usually localized to a specific brain area.

The estimated absorption changes at multiple wavelengths are then converted into hemodynamic changes (HbO and HbR) by applying the inverse transformation of the formulation of the modified Beer–Lambert law as follows:

(2) \begin{equation*} \left[\begin{array}{c}{\boldsymbol{x}}_{\lambda_1}\\{}\vdots \\{}{\boldsymbol{x}}_{\lambda_l}\end{array}\right]=\left[\begin{array}{cc}{\varepsilon}_{\mathrm{HbO}}\left({\lambda}_1\right)& {\varepsilon}_{\mathrm{HbR}}\left({\lambda}_1\right)\\{}\vdots & \vdots \\{}{\varepsilon}_{\mathrm{HbO}}\left({\lambda}_l\right)& {\varepsilon}_{\mathrm{HbR}}\left({\lambda}_l\right)\end{array}\right]\left[\begin{array}{c}{\boldsymbol{x}}_{\mathrm{HbO}}\\{}{\boldsymbol{x}}_{\mathrm{HbR}}\end{array}\right] \end{equation*}

where ${\boldsymbol{x}}_{\mathrm{HbO}}$ and ${\boldsymbol{x}}_{\mathrm{HbR}}$ are the concentration variations of HbO and HbR in the blood volume, respectively. The parameter ε is the molar extinction coefficient for each wavelength $\lambda$ and each Hb species. *l* is the number of wavelengths used for fNIRS measurement (in our study *l* = 3). ${\boldsymbol{x}}_{\lambda_1}\kern0.5em \cdots \kern0.5em {\boldsymbol{x}}_{\lambda_l}$ are the estimated absorption in the head volume at multiple wavelengths. In this study, the molar extinction coefficient values of each Hb species at wavelengths of 780, 805, and 830 nm were taken from the literature (Matcher et al. 1995).

### Forward Model Construction for DOT

To solve the inverse problem for 3D image reconstruction, we must establish forward modeling, that is, the computation of the sensitivity matrix **A** in equation (1). The forward modeling consists of 1) constructing a discrete 3D head model from anatomical image data, 2) coregistration of fNIRS optodes with the head model, 3) photon migration simulation, and 4) sensitivity computation of all voxels in the head model.

In this study, we constructed individual animals’ head models by segmenting their anatomical T1- and T2-weighted MR images into six optical layers (i.e., air, skull, cerebrospinal fluid, gray matter, white matter, and other soft tissues) using custom-made codes on MATLAB (MathWorks, USA). After the animals received a scalp incision surgery to affix optode sockets on their skull, we scanned the MR images again and generated the scalp-incised head model by replacing the superficial soft tissue layer into the air layer around the parietal region. The fNIRS optode positions were also coregistered to the scalp-incised head model.

The photon migration process inside the head tissue was simulated based on finite element modeling for discretized head models using the customized TOAST software (Koyama et al. 2005; Oki et al. 2009; Schweiger and Arridge 2014). In the photon migration simulation, tissue optical parameters (the coefficients of absorption and reduced scattering) for the wavelengths used in the recording (780, 805, and 830 nm) were taken from the literatures (Firbank et al. 1993; van der Zee et al. 1993; Simpson et al. 1998; Okada and Delpy 2003). A refractive index of *n* = 1.40 was used for all tissue layers. Then, we assigned wavelength and tissue-specific optical properties to all the voxels in the head models, and light propagation was simulated concerning the coregistered positions of optodes. Finally, the light transmission of each source–detector pair in the head volume, that is, spatial sensitivity profile of each optode pair, was obtained by calculating the photon measurement density function in each voxel and was normalized by the mean optical path length (Arridge and Schweiger 1995; Okada et al. 1995; Kawaguchi et al. 2007). We computed the spatial sensitivity profiles of all optode pairs to form sensitivity matrix **A** using the head model composed of 0.6-mm cubic voxels. As the sensitivity matrix **A** with low values renders the DOT calculation numerically unstable, we eliminated the voxels whose sensitivity values were smaller than a defined threshold (0.001 after normalizing the sensitivity). The normalized sensitivity was defined as the summation of sensitivity values across all channels divided by the maximum sensitivity value within the whole brain (gray matter). Supplementary Figures 2 and 3 in the Supplementary Material depict estimated spatial sensitivity profiles for each animal. The voxels in gray matter tissues ranging from frontal to parietal brain regions and from the brain surface to several millimeters in depth were selected for the subsequent DOT calculation.

### Image Reconstruction Algorithms

#### The Modified Minimum-Norm Method

We tested two algorithms for image reconstruction from the measured fNIRS data.

The first algorithm is the modified minimum-norm method (hereafter denoted as MN) (Hämäläinen and Ilmoniemi 1994). MN is one of the standard DOT methods formulated as an example of the regularization approach. It uses a regularization term consisting in the L2-norm of the solution under the assumption that the best solution is the one with minimum overall energy. The MN algorithm that we used minimizes a cost function as follows:

(3) \begin{equation*} \hat{\boldsymbol{x}}={\mathrm{argmin}}_{\boldsymbol{x}}\left({\left(\boldsymbol{y}-\mathbf{A}\boldsymbol{x}\right)}^{\boldsymbol{T}}{\boldsymbol{\Sigma}}^{-\mathbf{1}}\left(\boldsymbol{y}-\mathbf{A}\boldsymbol{x}\right)+\alpha{\left\Vert \boldsymbol{x}\right\Vert}^2\right) \end{equation*}

where $\boldsymbol{\Sigma}$ is the observation noise covariance matrix, computed from fNIRS data during pretask periods. More specifically, we computed the regularized noise covariance matrix using individual trial data to avoid instability and inaccuracy in the calculation of its inverse matrix as follows:

(4) \begin{equation*} \boldsymbol{\Sigma} =\tilde{\boldsymbol{\Sigma}}+\beta{s\mathbf{I}}_M \end{equation*}

(5) \begin{equation*} \tilde{\boldsymbol{\Sigma}}=\frac{1}{\mid{T}_{\mathrm{pre}}\mid}\sum_{v=1}^V\sum_{t\in{T}_{\mathrm{pre}}}{\boldsymbol{y}}_{tv}{\boldsymbol{y}}_{tv}^T \end{equation*}

(6) \begin{equation*} s=\frac{\mathrm{trace}\left(\tilde{\boldsymbol{\Sigma}}\right)}{M} \end{equation*}

where **I***_M_* is the *M × M* identity matrix. ${\boldsymbol{y}}_{tv}$ is the measured fNIRS data of all channels at time *t* of trial *v*, ${T}_{\mathrm{pre}}$ is a set of time points during the pretask period, and $\mid{T}_{\mathrm{pre}}\mid$ denotes the number of time points. $\beta$ is a regularization parameter.

The analytical solution of the optimization in equation (3) is

(7) \begin{equation*} \hat{\boldsymbol{x}}={\left({\mathbf{A}}^T{\boldsymbol{\Sigma}}^{-1}\mathbf{A}+\alpha{\mathbf{I}}_N\right)}^{-1}{\mathbf{A}}^T{\boldsymbol{\Sigma}}^{-\mathbf{1}}\boldsymbol{y} \end{equation*}

where **I***_N_* is the *N × N* identity matrix. For computing the MN solution, we obtained the optimal regularization parameter $\alpha$ by maximizing the marginal likelihood using 100 values in a logarithmic range, as implemented in our custom-made software (refer to the Supplemental Material for the details of this parameter search).

In the main experiments, we tested 6 different conditions of the MN algorithm as shown in Table 1. We controlled 1) the type of noise covariance matrix (full matrix or diagonal matrix), that is, whether to consider noise correlation between different channels or not, and 2) the regularization parameter $\beta$ of the noise covariance matrix, whose values were {1, 10^−2^, 10^−4^}. A larger $\beta$ indicates a larger modeling error in noise covariance estimation (refer to the Supplemental Material for how we set this parameter range). We hereafter designate each MN method of different conditions as MN201, MN202, and so on, respectively.

**Table 1:** The list of conditions tested for DOT image reconstruction using the MN algorithm

	MN201	MN202	MN203	MN204	MN205	MN206
Type of noise covariance matrix	Full	Full	Full	Diagonal	Diagonal	Diagonal
Regularization parameter $\beta$	1	10^−2^	10^−4^	1	10^−2^	10^−4^

*Notes*: We controlled 1) the type of noise covariance matrix (full or diagonal matrix) and 2) the regularization parameter $\beta$ of the noise covariance matrix.

#### The Hierarchical Variational Bayesian Method

The second DOT algorithm is the hierarchical variational Bayesian method (denoted as VB) that refines the solution of the MN algorithm using more detailed a priori information about brain activity. The VB algorithm that we tested (Shimokawa et al. 2012; Yamashita et al. 2016) is designed to separate explicit absorption changes in the scalp and those in the cortex with the combination of high-density measurements of fNIRS signals. However, because the signal contamination from scalp was eliminated in our data, we primarily focused on the parameters and conditions related to noise estimation and brain activity.

As a prior constraint about the cortical absorption changes ***x***, we assume that locally extended hemodynamic activation is represented by the sparse absorption changes ***z*** convolved with the spatial smoothing operator **W** as follows:

(8) \begin{equation*} \boldsymbol{x}=\mathbf{W}\boldsymbol{z} \end{equation*}

The *smoothing radius* parameter is the half-width of the Gaussian distribution of the spatial smoothing operator **W** that determines the minimal spread of brain activation.

Concerning the prior distribution $P(\boldsymbol{z})$, the VB algorithm incorporates the relevance parameters at each voxel that control the amplitude range of absorption changes in the corresponding voxels, that is, the prior belief in the spatial patterns of $P(\boldsymbol{z})$ (the initial value is given by an MN solution). The prior distribution of the relevance parameters is described as gamma distribution with shape parameter (${\gamma}_0$ in equation (8) in the study of (Yamashita et al. 2016)). As the shape parameter ${\gamma}_0$ regulates the confidence about the prior belief given as a form of relevance parameters, we hereafter refer ${\gamma}_0$ as “*prior confidence*.”

In the main experiment, we tested 48 different conditions by changing 1) the initial value, chosen from the 6 solutions of different MN methods in Table 1; 2) the smoothing radius parameter, either {0 or 2} mm; and 3) the prior confidence ${\gamma}_0$, whose values were {0, 10^−3^, 10^−2^, 10^−1^}. The estimated activation is expected to spread wider for larger smoothing radiuses. Additionally, higher ${\gamma}_0$ values lead to higher prior confidence. As ${\gamma}_0$ increases, VB estimation relies more on the initial estimation by the MN method. The type of noise covariance matrix and the regularization parameter $(\beta)$ were set as the same as those of the MN method used as the initial value. Table 2 describes the conditions that we tested using the VB algorithm. We hereafter refer to each VB method of different conditions as VB201, VB202, and so on. We iterated the calculation for maximizing free energy until its relative change with iteration was lower than 1e−10 (minimum iteration time was set as 500 times). We restricted the estimation of brain activity only within the voxels corresponding to gray matter tissues whose normalized sensitivity was >0.001. We updated the observation noise variance in the iteration process. We tested only two values as smoothing radius because much difference as far as the values of {2, 4, 8} mm was not observed in a pilot study (Supplementary Fig. 4 and Supplementary Tables 1–3 in the Supplementary Material).

**Table 2:** The lists of hyperparameters and conditions examined for DOT image reconstruction using the VB algorithm

**(a)**								
	**VB201 ~ 208**	**VB209 ~ 216**	**VB217 ~ 224**	**VB225 ~ 232**	**VB233 ~ 240**	**VB241 ~ 248**		
Initial value	MN201	MN202	MN203	MN204	MN205	MN206		
**(b)**								
***i* = [0,1,2,3,4,5]**	**VB201 + 8*i***	**VB202 + 8*i***	**VB203 + 8*i***	**VB204 + 8*i***	**VB205 + 8*i***	**VB206 + 8*i***	**VB207 + 8*i***	**VB208 + 8*i***
Smoothing radius (mm)	0	0	0	0	2	2	2	2
Prior confidence (${\gamma}_0$)	0	10^−3^	10^−2^	10^−1^	0	10^−3^	10^−2^	10^−1^

*Notes*: We controlled 1) six initial values, 2) two smoothing radius values, and 3) four prior confidence values. (a) The list of initial values used by each VB method. VB201–VB208 uses the solution of MN201 as their initial values but differ by their smoothing radius value and prior confidence value as listed in b. (b) The list of smoothing radius and prior confidence values used in each VB method. VB201 + 8*i* denotes the set of the methods of [VB201, VB209, VB217, VB225, VB233, and VB241] and VB202 + 8*i* denotes [VB202, VB210, VB218, VB226, VB234, and VB242], and so on. Methods in the same set differ by their initial values listed in (a).

### Statistical Analysis

We calculated the concentration changes of HbO and HbR in gray matter tissues using several DOT methods with different algorithms (MN and VB), and different hyperparameters and conditions. Then, we calculated the mean hemodynamic response change after the onset of the task ([0 10] s) of each voxel and statistically tested the difference between left-hand trials and right-hand trials using an unpaired two-sample *t*-test. In this manner, we obtained the 3D distributions of the *t*-values in gray matter tissues for each animal, Hb species, recording date and DOT method (“*t-*value images”). We calculated the *t*-values based on the left-hand trials relative to the right-hand trials. Thus, voxels showing positive *t*-values indicate that the voxels are activated more by left-hand trials than by right-hand trials (L > R), and voxels showing negative *t*-values indicate vice versa (L < R). We adopted the max *t* method to determine significantly activated voxels to correct the family-wise error rate in multiple comparison tests (Nichols and Hayasaka 2003); we randomly assigned data of individual trials as one of two labels, and *t*-values were calculated for each voxel using the randomly assigned labels 2000 times. The threshold of *t*-values was determined as the top 0.5% highest *t*-value of this permutation test. Finally, the voxels whose *t*-values exceeded the threshold were extracted as the areas significantly activated at a significance level of 0.5% by left-hand or right-hand tasks (“activated-area map”).

### Reliability Evaluation of DOT Reconstruction

We quantitatively evaluated the reliability of DOT reconstruction using several metrics. First, we classified all voxels into “significant (L > R) activation,” “significant (L < R) activation,” or “No significant activation,” based on the *t*-values calculated from single-day data. We then quantified the consistency of the classification across dates by calculating Fleiss’s kappa (Fleiss 1971) and Gwet’s AC1 coefficient (Gwet 2008a, 2008b); we assumed the 3-class classification results for *N* voxels from *D* dates, as the 3-class rating results for *N* samples by *D* raters and calculated the agreement of rating results across raters as Fleiss’s kappa. Gwet’s AC1 coefficient is a similar agreement index as Fleiss’s kappa, while mitigating the vulnerability to a certain imbalance in the rating results (Wongpakaran et al. 2013). We calculated the mean Dice coefficient as the third measure; we extracted the voxels of “L > R” label and “L < R” label and calculated the similarity of classification results between all pairs of different dates. We also calculated the mean detection rate (the rate of voxels showing significant task modulation of all interested voxels) based on the single-day data to evaluate the constancy of DOT reconstruction across dates. Regarding other metrics, we segmented *t*-value images into several ROIs (see next section for the details) and calculated intraclass correlation coefficient (1,1) (McGraw and Wong 1996). Specifically, we evaluated the difference between the mean *t*-values from two ROIs with respect to the variance across different dates as a way to assess the reproducibility of DOT image reconstruction.

### Time-Series Plot of HbO and HbR Changes in Individual Brain Areas

We performed regional parcellation by applying the nonlinear alignment of a digital anatomical atlas (D99 atlas (Reveley et al. 2017)) to the brain images of individual animals using the AFNI analysis package (Cox and Jesmanowicz 1999). Next, we manually merged areas, especially small areas, based on coarser anatomical taxonomy. However, the primary motor area (F1) was manually parcellated into the hand area (the medial bank of the central sulcus and the neighboring gyrus at the rostrocaudal levels through the superior genu of the central sulcus) and the other area. Finally, we selected 44 brain areas whose area size was larger than 50 voxels in both animals for further ROI-based analysis.

We averaged the HbO/HbR changes within the voxels of each brain area after DOT reconstruction. We then calculated the trial-averaged HbO/HbR changes for left-hand and right-hand tasks. To visualize the bilateral difference in the time plot of HbO and HbR changes in individual brain areas, we subtracted the time series data of HbO/HbR changes for right-hand tasks from those for left-hand tasks and calculated the confidence interval of these L-R time-series data using a bootstrap procedure (repeat time = 1000). We interpreted the increase in HbO changes and the decrease in HbR changes in the brain areas contralateral to the hand movement as “contralateral hand modulation.” The peaks of the contralateral hand modulation were defined by maximum or minimum of the L-R time-series data within the time window of [0 10] s. The brain areas whose modulation peak was 10 times smaller than the maximum modulation peak in the animal were eliminated from the analysis after peak detection. We also eliminated the frontal areas in the left hemisphere from the analysis because no contralateral hand modulation was observed.

## Results

### 3D Activated-Area Map

We investigated two different DOT algorithms (MN and VB) by systematically manipulating hyperparameters and conditions to reconstruct the 3D images of HbO and HbR changes in the brain volume from the fNIRS signals recorded from the skull surfaces of monkeys. We observed that the DOT algorithms stably calculated the images and the value of algorithm’s objective function (free energy) monotonically converged within several iterations. We obtained the reconstructed 3D images of HbO and HbR changes with the voxel size of 0.6-mm cubic voxels (refer to Supplementary Figs 5 and 6 in the Supplementary Material). The resolution of DOT images reported in this study was higher than that of our previous human DOT study; the voxel size of the reconstructed images using the same DOT algorithm was 4-mm cubic voxels in the previous study (Yamashita et al. 2016), in which the optical signals were recorded from the scalp surfaces of human subjects with a probe interval of 13 mm.

**Figure 2:**
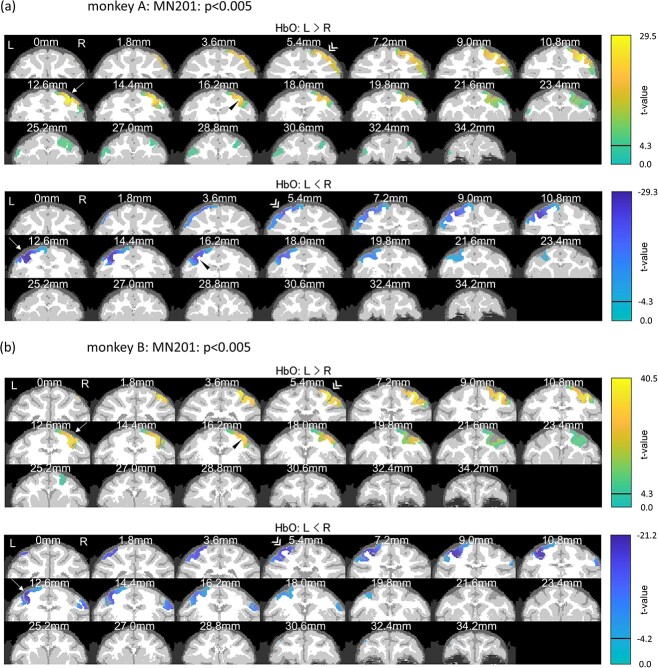
Color map of *t*-values (*activated-area map*) for HbO that exceeded the significance level of 0.5%. Plots are based on the reconstruction results using the MN201 method after merging data across dates. T-value map was overlaid on the coronal sections of the head model images. The numbers indicate the distance of each coronal section from the auditory canal in the anterior–posterior axis (all sections are anterior). (a) T-value map for monkey A. (b) T-value map for monkey B. Upper panels in (a) and (b) depict the brain areas that are significantly more activated by left-hand tasks than by right-hand tasks (L > R), and the lower panels depict vice versa (L < R). Significant cortical hemodynamic modulation was revealed around the upper limb regions of the primary motor area, especially in the anterior bank of the central sulcus (white arrows), as well as the premotor (black arrowheads) and parietal areas (white double arrowheads) contralateral to the hand movement in both animals.

We next statistically analyzed the hemodynamic response changes between left-hand and right-hand trials to identify the voxels that demonstrated significant modulation depending on the side of the hand used in the task. We merged the fNIRS data recorded on different days within each animal and applied a *t*-test to the reconstructed images of HbO and HbR changes, respectively. Positive *t*-values indicated that the voxels demonstrated larger Hb change in the left-hand task than in the right-hand task, and negative *t*-values indicated vice versa. Figure 2 depicts the color maps of *t*-values for HbO, overlaid on the head model sectioned in coronal planes (for HbR, refer to Supplementary Fig. 7 in the Supplementary Material). The voxels whose *t*-values exceeded 0.5% significance level are shown in the figure. The results revealed significant cerebral hemodynamic modulation around the upper limb regions of the primary motor area, especially in the anterior bank of the central sulcus (white arrows in Fig. 2), as well as the premotor (black arrowheads) and parietal areas (white double arrowheads) contralateral to the hand movement in both animals.

In contrast, weak but significant activity was also observed at the frontal areas in the ipsilateral hemisphere (L > R in monkey A and L < R in monkey B). When we defined the “*core activated areas”* as voxels whose *t*-values exceeded the criteria of the top 5% highest and lowest *t*-values (Supplementary Fig. 8 in the Supplementary Material), the areas were limited more to the contralateral hemisphere (refer to Supplementary Figs 9 and 10 in the Supplementary Material for the detailed inspection of core activated areas).

### Evaluation of the DOT Reconstruction

**Figure 3:**
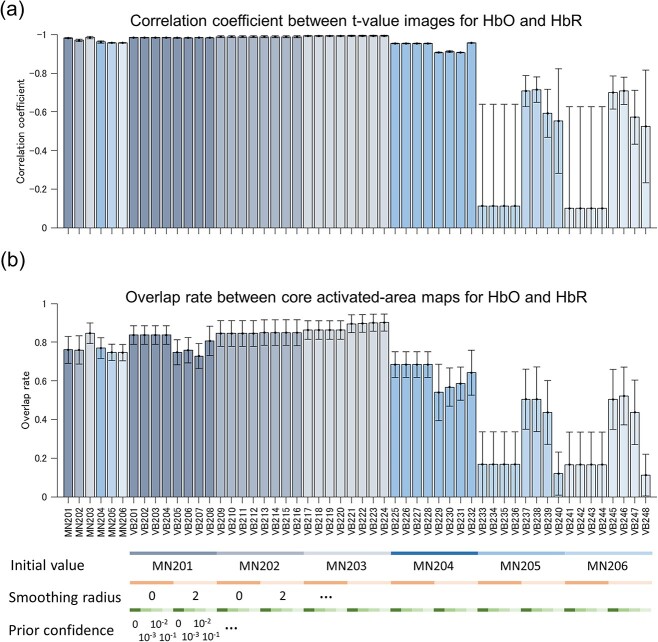
Comparison between image reconstruction results from different DOT methods. *X*-axis labels indicate the DOT methods used. Error bars indicate the SE. *T*-value images were calculated from the data merged across different recording dates in this analysis. (a) Mean correlation coefficient between *t*-value images for HbO and HbR. Data were averaged over two animals. (b) Overlap rate between core activated-area maps for HbO and HbR. Core activated areas were defined by voxels that exceeded the criteria of the top 5% highest and lowest *t*-values as depicted in Supplementary Fig. 8 in the Supplementary Material. Data were averaged over the two animals, Hb species (HbO and HbR), and tasks (L > R and L < R).

It is considered that the increase in regional cerebral blood flow in response to neural activity produces an increase in HbO and a decrease in HbR. Therefore, reliable DOT reconstruction should yield a negative correlation between the *t*-value images for HbO and HbR. It is important to note that the activated-area maps for HbO and HbR were nearly identical with a sign reversal, thereby supporting the reliability of DOT reconstruction (Fig. 2 and Supplementary Fig. 7). Figure 3*a* shows that the correlation coefficients between the *t*-value images for HbO and HbR are close to −1, except for the VB methods using the diagonal noise covariance matrix (VB225–VB248). We also calculated the overlap rate between the core activated-area maps for HbO and HbR (maps in Supplementary Figs 9 and 10 in the Supplementary Material), as shown in Figure 3*b*. Again, all DOT methods, except for the VB methods using the diagonal noise covariance matrix, demonstrated a high overlap rate.

**Figure 4:**
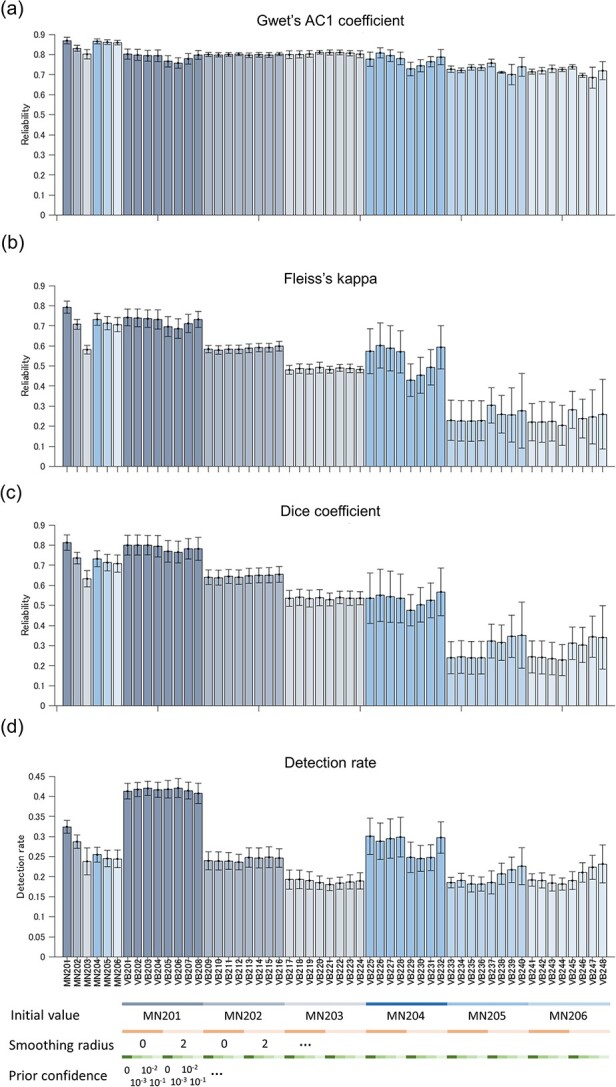
Reliability measures and detection rate of different DOT methods (voxel-based single-date analysis). *X*-axis labels indicate the DOT methods used. Mean values were calculated across the two animals and Hb species for all metrics. Error bars indicate the SE. (a) Gwet’s AC1 coefficient. (b) Fleiss’s kappa. (c) Dice coefficient. (d) Detection rate.

To evaluate the reliability of different DOT methods in another manner, we next calculated the consistency of the reconstruction results across recording dates using several metrics as described in methods. Figure 4*a*–*d* shows the results of reliability evaluation using Gwet’s AC1 coefficient, Fleiss’s kappa, Dice coefficient, and detection rate. MN201 and the VB methods that used the MN201 solution as the initial values (VB201–208) provided relatively high reliability scores and detection rates compared with other methods in all measures. Three-way ANOVA conducted on the results of the VB methods for these four metrics revealed significant main effect of “initial value” (=choice of hyperparameters related to the MN algorithm) for all four measures (Supplementary Table 4 in the Supplementary Material). These results indicate that appropriate regularization parameter ($\beta =1$) and noise covariance calculation, including interchannel noise correlation, are critical for obtaining reliable DOT reconstruction for consistency across dates. Fleiss’ kappa is >0.6 in our best MN and VB methods, indicating that the agreement of the task-dependent activation pattern across dates is substantial. To investigate the effect of additional prior information dedicated to the VB algorithm, we conducted a many-to-one comparison using Dennett’s test between MN201 and the VB methods using the MN201 solution as the initial value. The test result did not reveal any significant difference, except for the detection rate measure (Supplementary Table 5 in the Supplementary Material), indicating that the VB algorithm did not overcome the MN algorithm in our experiment.

### ROI-Based Analysis

**Figure 5:**
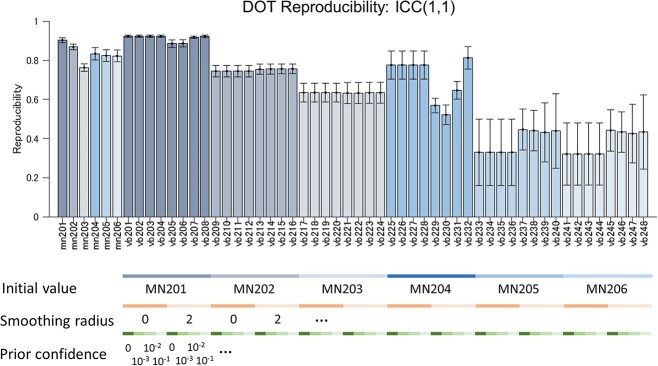
Intraclass correlation coefficients (1,1) between *t*-value images from different DOT methods (ROI-based single-date analysis). *X*-axis labels indicate the DOT methods used. Mean values were calculated across the two animals and Hb species. Error bars indicate the SE.

The ROI-based analysis (analysis based on individual brain areas) of the HbO images reconstructed using the MN201 method showed a core in the task-modulated activation (top 5% voxels, Supplementary Fig. 9 in the Supplementary Material) at intraparietal areas (VIP, LIPv, LIPd, and area 7a), area 7b, area 5, primary somatosensory area (areas 1–2), area 3a/b, primary motor area (F1), dorsal premotor area (F2), and ventral premotor area (F4) in the hemisphere contralateral to the hand used in the tasks commonly in both animals. Monkey B additionally showed significant core activation of L > R at right AIP. Most of the above-listed areas were constantly identified as core activated areas using the MN and VB201–VB208 methods. We also calculated the maximum and minimum *t*-values of 44 brain areas as an intensity measure of task modulation in individual brain areas. The average correlation coefficients between ROI-wised task modulation measures of every different 2 days were high (correlation coefficient > 0.9) for MN201 and VB201–V208 (Supplementary Fig. 11 in the Supplementary Material). Figure 5 shows the intraclass correlation coefficients (1,1) from *t*-value images segmented into ROIs. The reproducibility of DOT reconstruction defined by this measure also produced a high correlation coefficient when using MN201 and VB201–V208 (refer to Supplementary Fig. 12 in the Supplementary Material for the voxel-based intraclass correlation coefficient (Shou et al. 2013) between left-hand and right-hand trials using the original DOT images). These findings indicated the constancy of DOT reconstruction across dates in terms of ROI-based analysis as well as voxel-based analysis.

**Figure 6:**
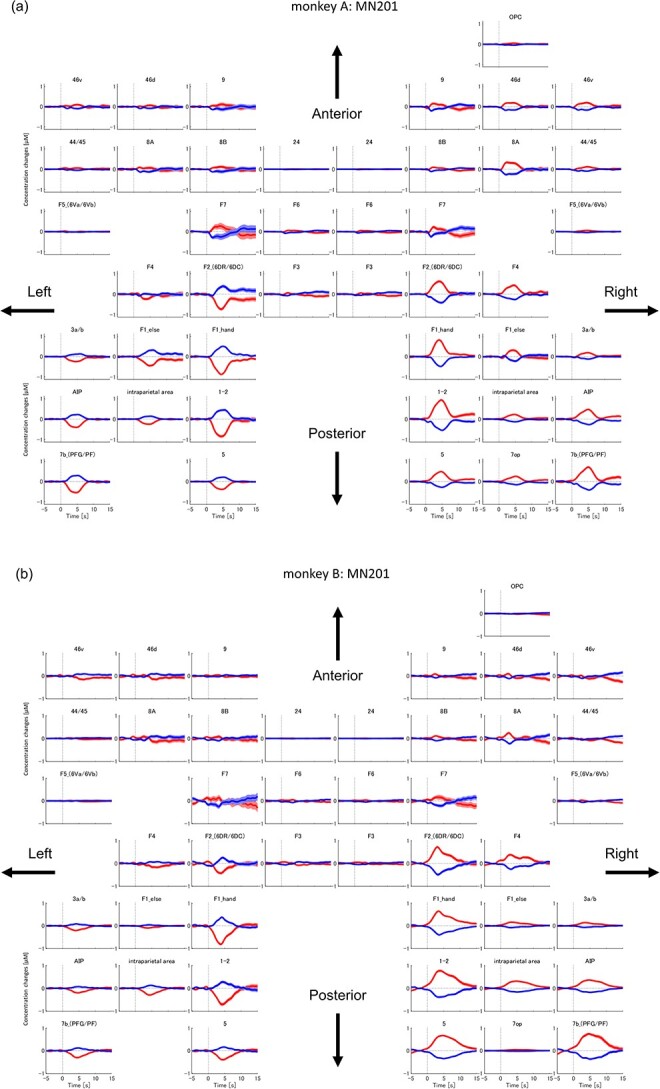
Trial-averaged time plot of HbO/HbR changes in each brain area estimated using the MN201 method. Red lines indicate the mean L-R HbO changes (left-hand trials minus right-hand trials), and blue lines indicate the mean L-R HbR changes. Shaded areas represent a 95% confidence interval estimated by permutation test (repeat time = 1000). The areas in the right hemisphere show increment in L-R HbO changes and decrement in L-R HbR changes, whereas the areas in the left hemisphere show vice versa. (a) Time plots for monkey A. (b) Time plots for monkey B.

**Figure 7:**
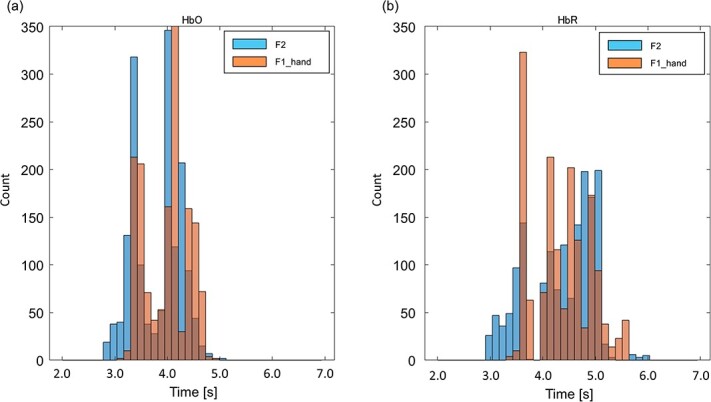
Histogram of peak latency of top 300 voxels in the dorsal premotor area (F2) and the primary motor hand area (F1 hand area). Blue bars indicate the frequency distribution of peak latencies in the F2, and red bars indicate those in the F1 hand area. (a) Histogram from HbO responses. (b) Histogram from HbR responses.

Figure 6 depicts how HbO and HbR concentrations changed over time in each brain area for each animal. We subtracted the mean HbO/HbR signals of right-hand trials from those of left-hand trials (L-R changes) and plotted them as red/blue lines, respectively. The areas in the right hemisphere show increment in the L-R HbO changes and decrement in the L-R HbR changes. In contrast, the areas in the left hemisphere show vice versa, indicating how contralateral hand modulations changed over time. Cross-correlation analysis between the time-series of HbO/HbR changes in the listed 44 areas (Supplementary Fig. 13 in the Supplementary Material) indicated that the cortical areas were roughly divided into three regions in terms of activation timing, viz., 1) frontal areas (around area 8, area 9, and area 46), 2) premotor (F2 and F4) and primary motor (F1 hand area) areas, and 3) primary somatosensory (1–2) and parietal areas (area 3, AIP, area 5, area 7). The peak latencies of contralateral hand modulation were earliest in the right frontal areas (2.89 $\pm$0.989 s (mean ± standard deviation, SD)), followed by premotor and primary motor areas (4.17 $\pm$0.833 s), and then somatosensory and parietal areas (4.78 $\pm$0.389 s) in both hemispheres. The main effect of regional difference on peak latency was significant (one-way ANOVA: *F* = 50.13, df = 81, *P* ≪ 0.001), and multiple comparisons showed that the peak latencies of the three regions were significantly different from each other. We also compared the peak latency for trial-averaged HbO/HbR changes of individual voxels within the dorsal premotor area (F2) and primary motor hand area (F1 hand area). The mean peak latencies of top 300 voxels of largest modulation in HbO change in the dorsal premotor area and those in the primary motor hand area were 3.76 $\pm$0.437 s (mean ± SD) and 3.94 $\pm$0.410 s, respectively (the mean peak latencies for changes in HbR were 4.30 $\pm$0.612 and 4.32 $\pm$0.546 s, respectively). Figure 7 shows histograms of individual voxels’ peak latencies for HbO and HbR. The nonparametric Wilcoxon rank-sum test showed that the difference in HbO peak latency was significant (*z* value = −10.9565, rank-sum = 1 256 082, *P* ≪ 0.001). Although some voxels in F2 showed an earlier HbR peak latency than what was observed for any voxel in the F1 hand area, we did not find any significant differences in overall HbR peak latency between the two areas.

We also observed the overall contralateral hand modulation even in the time plot of single-trial data (Supplementary Fig. 14 in the Supplementary Material). These results demonstrate the potential of our fNIRS–DOT protocol to analyze the brain activity changes in each cortical area at a sampling rate as high as 7.69 Hz.

## Discussion

Our study demonstrated that oxygenated and deoxygenated Hb changes in the cortical blood volume could be reconstructed as 3D images by DOT with a relatively high sampling rate and fine voxel size. In contrast to our previous monkey fNIRS studies, the present study localized the activity changes in gray matter tissues along the central sulcus. According to the previous histological study, the primary motor area has two subdivisions. One is an evolutionarily old rostral region that lacks corticomotoneuronal cells. The other is a caudal region located along the anterior bank of the central sulcus. The latter is present only in some higher primates and humans and contains shoulder, elbow, and finger corticomotoneuronal cells (Rathelot and Strick 2009). An fNIRS–DOT technology that can identify activation profiles within the sulcus could reveal a more detailed picture of brain activity related to dexterous hand movements than can be obtained using conventional fNIRS topography.

Our study results showed that contralateral hand modulation appeared first in frontal areas, followed by premotor to primary motor areas, and then in parietal areas. We also found that the peak latency for HbO changes in the voxels within the dorsal premotor area preceded those within the primary motor area. This finding is consistent with previous physiological studies showing that the single-unit activity recorded in the dorsal premotor area precedes the unit activity in the primary motor area (Cisek et al. 2003; Umilta et al. 2007). Therefore, the fNIRS–DOT protocol demonstrated in our study is a promising approach to explore the dynamics of networks through ROI-based time-series analysis while the animal behaves freely, a situation where other imaging technologies, such as fMRI and PET, cannot be used. A method that enables the recording of hemodynamic response signals at a high sampling rate will be useful for causality analysis or other time-series analyses.

We note several explanations for why we achieved good DOT reconstruction results. In our experimental system, optodes were directly affixed to the skull, which likely reduced any motion artifacts. Additionally, fNIRS signals in our system are not contaminated by scalp blood flow, which is a serious problem for usual DOT reconstruction. Furthermore, we benefited from using monkeys who have smaller heads than humans, which led to a better SNR than has been possible in previous human studies.

Developing better brain imaging techniques for animal experiments has its own significance because the techniques can be used together with other invasive experimental techniques such as electrode recording, drug/virus injection, and/or cortical lesioning. In fact, we demonstrated that our fNIRS system was valuable to investigate the brain function changes after the surgically introduced lesion of the primary motor cortex, which revealed the cortical areas that were newly recruited for the recovery of hand movements after the legion (Kato et al. 2020). Studies using fNIRS on animals combined with other invasive techniques would also be critical to clarify the relationship between the hemodynamic responses and the underlying neural mechanism, whose relationship is not yet completely uncovered (Logothetis et al. 2001; Sheth et al. 2004). We can also expect to accelerate translational research from invasive monkey fNIRS studies to noninvasive human fNIRS studies.

Nonhuman primate research is critical in examining the neural substrate of complex cognitive and/or motor functions as human functions (Courtine et al. 2007). We selected macaque monkeys as an animal model for our research on hand movement control because these monkeys have a highly developed dexterous hand function that is similar to that in humans (Isa et al. 2007; Lemon 2008). The homology between humans and macaques is also observed in the visual system (Kiani et al. 2007; Kriegeskorte et al. 2008). Hence, the significance of our proposed fNIRS–DOT protocol is not limited to the study of motor function alone. It also applies to visual function and the multimodal interaction between them. We confirmed that fNIRS recording from the skull surface above the visual cortex, where we electrophysiologically measured the unit activity in response to visual object images (Hayashi and Kawata 2018), can provide visually evoked signals with a high signal-to-noise ratio as well.

Our results demonstrated that reconstructed HbO images negatively correlated with HbR images, and the maps of statistically significant HbO/HbR changes corresponded very well with each other. The strong negative correlation between HbO and HbR changes was observed only in previous animal experiments conducted using skull-exposed rodents (Berwick et al. 2002; Sheth et al. 2004; Dunn et al. 2005). Our results provide further support for the negative coupling of HbO and HbR in the cortex, on which coupling some hemodynamic signal separation methods for noise reduction (Yamada et al. 2012; Cui et al. 2010) relies.

Our study also provided several insights into the optimal configuration of DOT algorithm using real experimental data without major signal contaminations or artifacts. After systematically exploring the hyperparameters and conditions in the DOT process, we confirmed that the reconstruction results are more reliable when using individual trial data than trial-averaged data (Supplementary Fig. 4 in the Supplementary Material) probably because animal behavior and hemodynamic change fluctuate trial by trail. In addition, the trial data could allow for more reliable noise covariance estimation than trial-averaged data, as the number of data samples is much larger. It is also very critical to optimize the regularization parameter of the noise covariance matrix, and the covariance calculation should include interchannel noise correlation. However, the VB methods provided reliable results irrespective of hyperparameters about the minimal spread of brain activity (smoothing radius) and the prior confidence parameter when DOT calculation started from a near-optimal solution provided by an MN method. We did not observe any clear advantage of the VB algorithm over the MN algorithm in the present study. The VB algorithm would provide finer source separation if we measured dense fNIRS signals with more optical path overlaps to make available spatial information about the underling brain activity redundant for the source estimation (Shimokawa et al. 2012; Yamashita et al. 2016).

Although the voxel size of the reconstructed image was as fine as 0.6-mm cubic-voxel in our study, it does not mean that the activation foci can be distinguished with as high spatial resolution as the image resolution. Nevertheless, image reconstruction with a fine voxel size is critical for accurate ROI-based analysis. It is the future study to examine whether we can distinguish different functional brain areas such as the hand or the elbow area within the same hemisphere using our protocol.

If fNIRS recording were phase-locked at a fixed period, then the signal would be affected by underlying low-frequency oscillations (e.g., Mayer waves around every 10 s). Although we provided the food pellets approximately every 20 s, fNIRS data were taken when the monkeys were voluntarily moving their hands. In addition, the Mayer wave component in the fNIRS signal is supposed to originate from the myogenic activity of vessels and is generally observed from all channels only in HbO. In contrast, our experimental data showed clear negative coupling between HbO and HbR in the brain areas contralateral to the hand used in the reaching task. Therefore, we concluded that the contribution of Mayer waves to our observations was minor.

The proposed fNIRS–DOT protocol requires an invasive operation that is currently inapplicable to human studies. However, the fNIRS optodes were assembled with LEDs and photodetectors aligned on a flexible substrate (Yamakawa et al. 2019) and the chips of LEDs and photodetectors can be technologically miniaturized to a submillimeter scale. Therefore, it would be feasible to implant such miniaturized probes under the scalp in a relatively less invasive manner in future and to use our fNIRS–DOT protocol for obtaining high-resolution 3D functional brain imaging data from human participants under naturally behaving situations. The critical factors for reliable DOT image reconstruction and the methodology used to evaluate the reliability of the reconstruction results demonstrated in our study would be informative even in such a future human fNIRS study considering the homology between human and nonhuman primates in terms of anatomical and functional brain structures.

In summary, we demonstrated for the first time that DOT could provide reliable 3D functional brain images with a fine voxel size when using the fNIRS signals recorded directly from the monkey’s skull surface. We also showed that significant activation was constantly observed at the motor-related areas, including areas in the central sulcus, contralateral to the hand movement, although fNIRS data were recorded under head-free conditions. In contrast to fMRI and PET, our fNIRS–DOT protocol allowed us to investigate the timing of hemodynamic response changes in individual brain areas at high sampling rate (>7.69 Hz).

## Author contributions

All authors contributed to the entire designing of this research. R.H. led the project, wrote the manuscript, and performed the ROI-based analysis to validate the DOT algorithms from a physiological viewpoint. O.Y. performed the DOT reconstruction, designed the framework of parameter search and statistical tests, and investigated the reliability of the DOT algorithm. H.K. constructed the head model and performed photon migration simulation and ROI segmentation. T.Y. contributed to collecting the fNIRS and anatomical MRI data, and designing the fNIRS recording system. N.H. contributed to the designing of animal experiments and physiological interpretation of the results. All coauthors contributed to improving the quality of the manuscript.

## Funding

JSPS KAKENHI (20H04597, 19H04200, 18H05019); JST Moonshot R&D (JPMJMS2012); AMED Research & Development Program.

## Notes

*Conflict of Interest*: The authors declare no conflict of interest.

## Data availability

Data reported in this manuscript are available from the corresponding author on reasonable request.

## Supplementary Material

Supplementary_material_Hayashi_cerebral_cortex_communication_final_tgab064
