# Supplementary material for: Diffuse Optical Tomography Using fNIRS Signals Measured from the Skull Surface of the Macaque Monkey
Source: Cereb Cortex Commun. 2021 Nov 10;3(1):tgab064. doi: 10.1093/texcom/tgab064 (PMC8767783; doi:10.1093/texcom/tgab064)
Supplement: Supplementary_material_Hayashi_cerebral_cortex_communication_final_tgab064 [file supplementary_material_hayashi_cerebral_cortex_communication_final_tgab064.zip › Supplementary_material_Hayashi_cerebral_cortex_communication_final_tgab064.docx]

**Diffuse optical tomography using fNIRS signals measured from the skull surface of the macaque monkey**

Ryusuke Hayashi, Okito Yamashita, Toru Yamada, Hiroshi Kawaguchi, Noriyuki Higo

**Supplementary material**

**Optode positions of each animal**


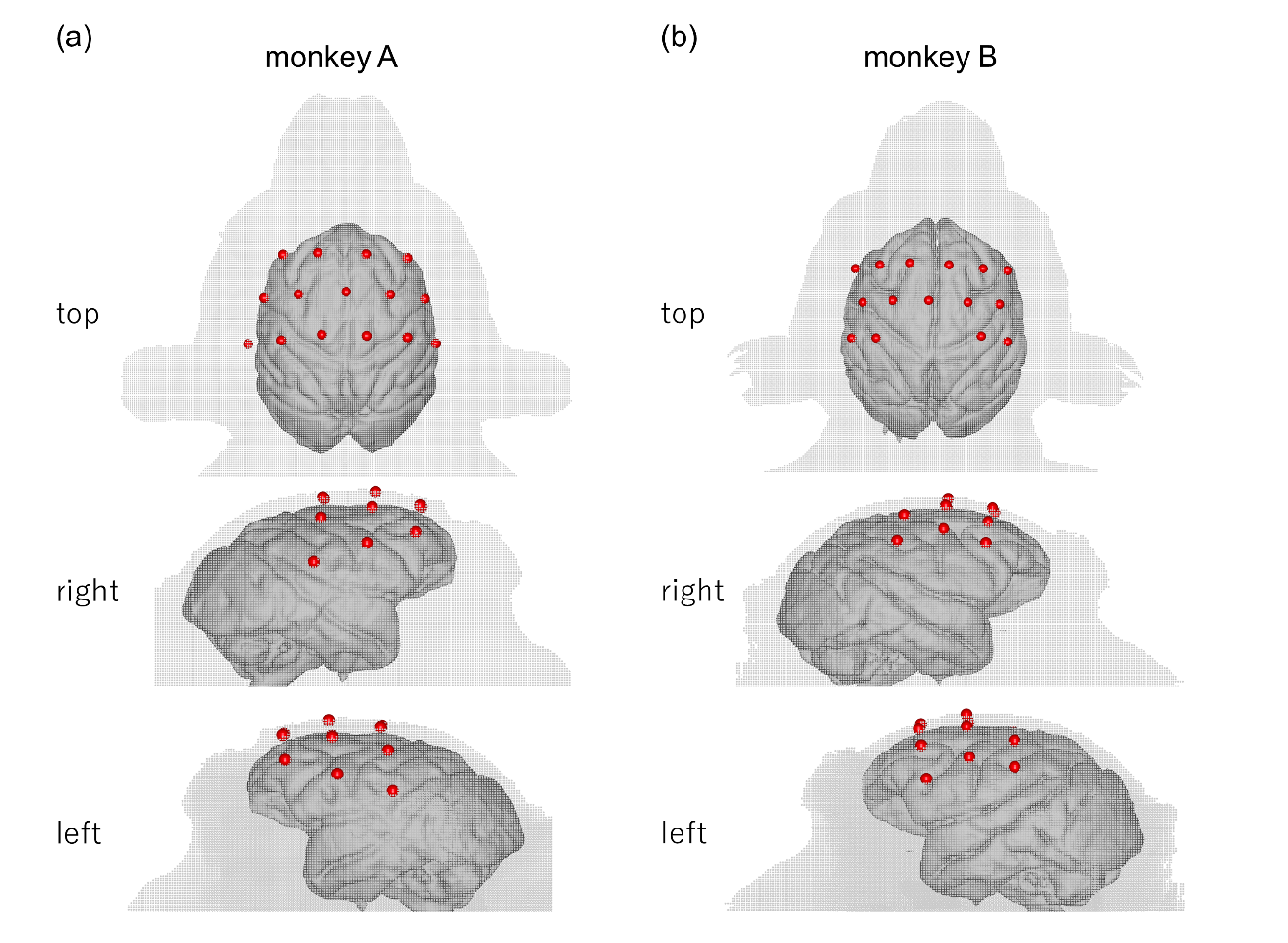


**Figure S1.** fNIRS optode positions with respect to the cortical surface of monkey A (a) and monkey B (b). Red spheres indicate the positions of the optode tips. Top, middle, and bottom rows are the top-, right-, and left-view images.

**Spatial sensitivity profile of each animal**


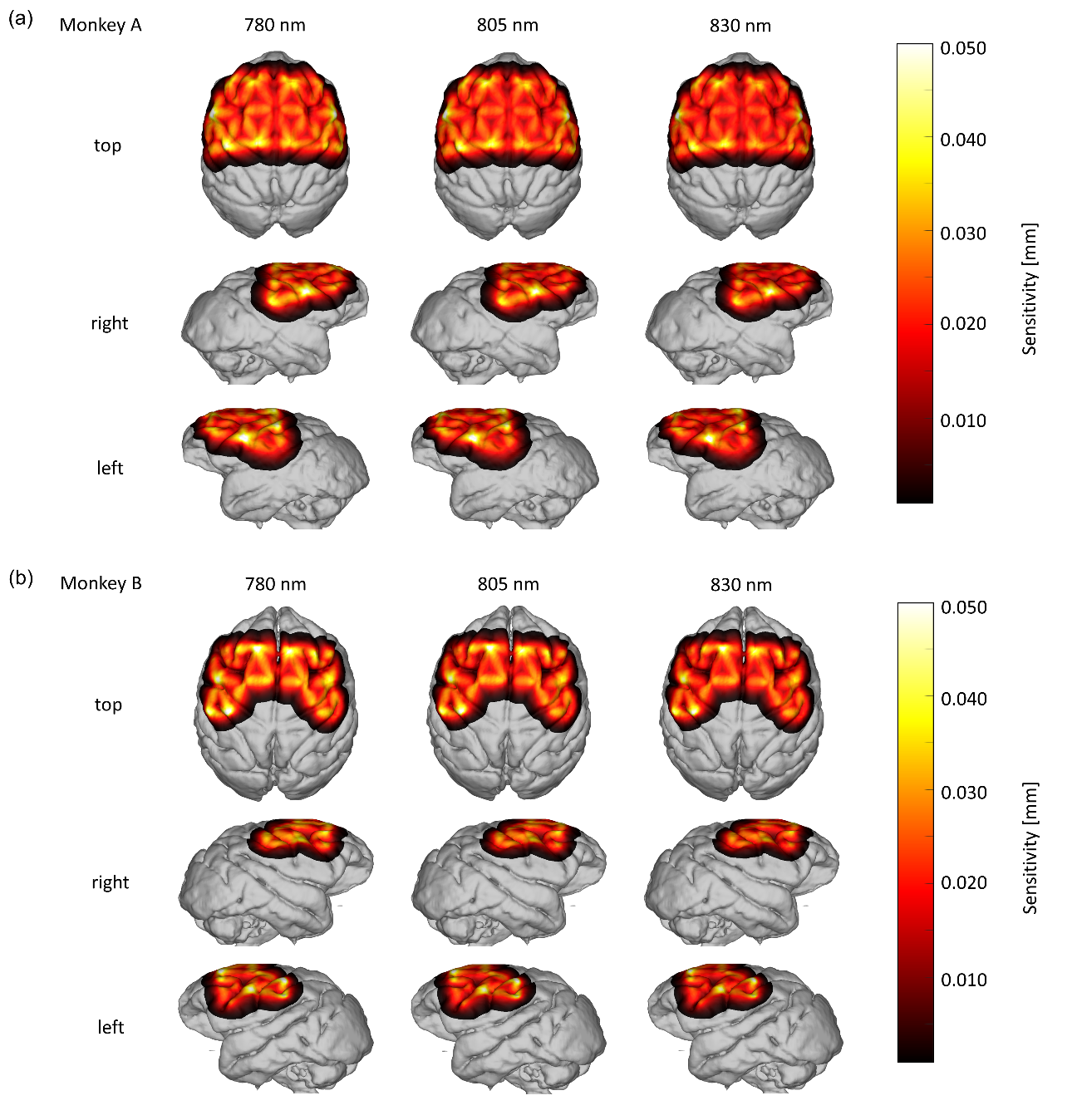


**Figure S2.** Estimated spatial sensitivity profiles for monkey A (a) and monkey B (b) at three wavelengths. The maximal sensitivity values across all channels are depicted as color maps overlaid on the cortical surfaces.


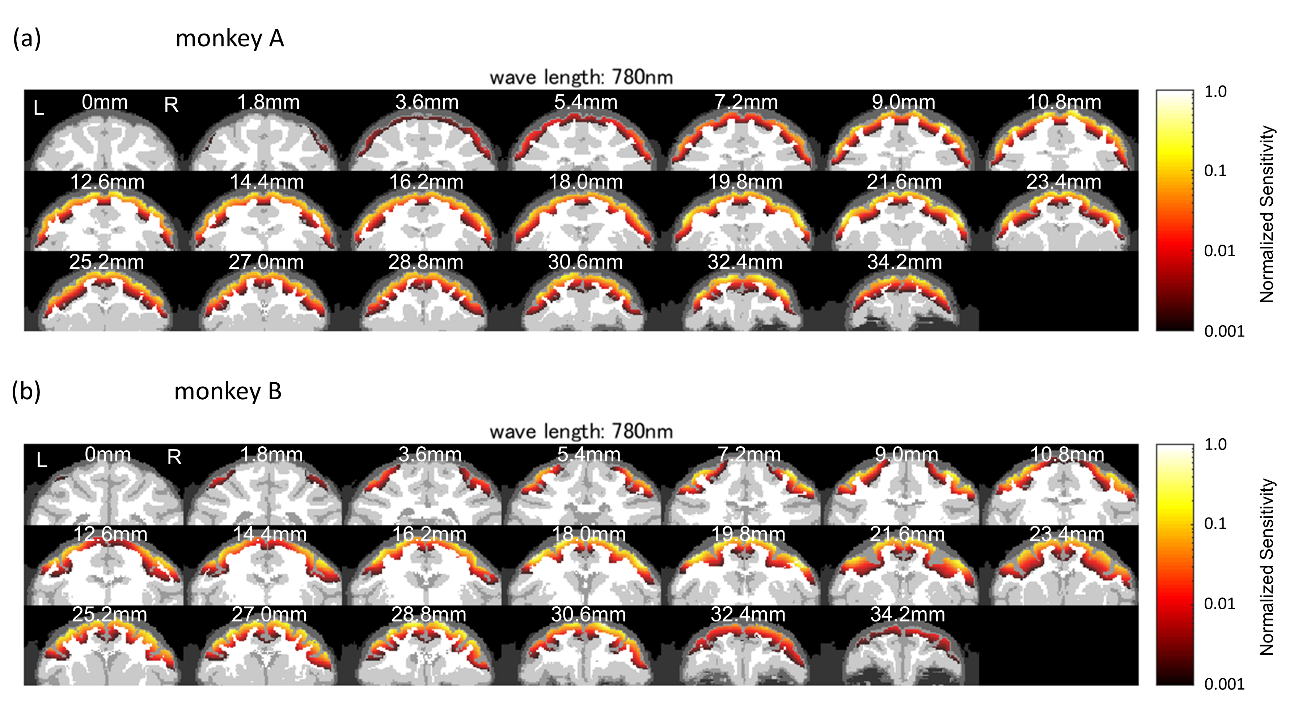


**Figure S3.** Estimated spatial sensitivity profiles for monkey A (a) and monkey B (b) at a wavelength of 780 nm. The normalized sensitivity values are depicted as color maps overlaid on the coronal sections of the head model images. The numbers indicate the distance of each coronal section from the auditory canal in the anterior-posterior axis (all sections are anterior). The voxels corresponding to gray matter tissue in which normalized sensitivity was > 0.001 were reconstructed by DOT.

**Parameter search via the MN method**

We optimized regularization parameter$\alpha$ in the MN method using the following grid-search configuration. First, we set the minimum and maximum singular values of the sensitivity matrix **A** as $s_{min}$ and $s_{max}$. Then, we let $\xi_{min}=log(s_{min})-5$ and $\xi_{max}=log(s_{max})-5$, and divided the search values as follows.

$$\xi\left( n \right)=\xi_{min}+n\times\frac{{(\xi}_{max}-\xi_{min})}{99}$$

Where n = {0, 1, …, 99}.

Finally, we set $\alpha\left( n \right)=exp(\xi\left( n \right))$ and calculated the best regularization parameter$\alpha$ that maximized the marginal likelihood.

In our previous human DOT study (Yamashita et al., 2016), the regularization parameter 𝛽 of the noise covariance matrix was optimal in the range of [1, 4], which excluded optode coregistration errors as modeling errors. Because optode positions were fixed in the present monkey DOT study, and because optode coregistration errors are thought to be smaller here than in the previous human study, we set the regularization parameter 𝛽 as {1, 10^-2^, 10^-4^} for the parameter search.

**Pilot study of parameter search for DOT reconstruction**

As a pilot study, we first tested three different conditions for DOT image reconstruction using an MN algorithm, as shown in Table S1. We controlled 1) two different modes for calculating noise covariance matrix and 2) whether including modeling error or not in the DOT process. The meaning of the covariance matrix modes is depicted in Table S2. The modes differ depending on i) whether the covariance matrix is calculated from trial-averaged data or single-trial data and ii) whether the observation noise covariance matrix is a diagonal matrix or a full matrix. In contrast to the MN algorithm used in the main experiment, we simply considered modeling error rates in the pilot study rather than using regularization parameters. The weight of the modeling error is set as 0.002 if any. We hereafter refer to each MN method of different conditions as MN01, MN02, and MN03, respectively.

**Table S1.** The list of different conditions tested for DOT image reconstruction using the MN algorithm. We controlled 1) covariance matrix mode and 2) modeling error weight. Table S2 shows the meaning of each covariance matrix mode.

| **MN** | **01** | **02** | **03** |
| --- | --- | --- | --- |
| **Covariance matrix mode** | A | B | B |
| **Modeling error weight** | 0.002 | 0.002 | 0 |

**Table S2.** The list of covariance matrix modes. We used three different modes to estimate the covariance matrixes of observation noises by changing i) whether using the trial-averaged data or single-trial data and ii) whether the noise covariance matrix is a diagonal matrix or a full matrix.

| **Covariance matrix mode** | **Data used for calculation** | **Type of noise covariance matrix** |
| --- | --- | --- |
| A | Trial-averaged data | Diagonal covariance matrix |
| B | Single-trial data | Full covariance matrix |
| C | Single-trial data | Diagonal covariance matrix |

We also tested several different conditions for DOT reconstruction using the VB algorithm by changing a) the modes for estimating noise covariance matrix, b) the modeling error weight, c) the minimal size of brain activity (smoothing radius) {0.1, 2, 4, 8} mm, d) whether updating observation noise variance or not in the DOT process, and e) the initial values. Table S3 describes the conditions that we tested for DOT reconstruction using the VB algorithm. We refer to each VB method of different conditions as VB00, VB10, and so on. We further distinguish the VB methods using different initial values, i.e., the solution of different MN methods as VB00-MN01, VB00-MN02, and so on.

We iterated the algorithm until the relative change of the free energy was lower than 1e-4. We set the prior confidence parameter $\gamma_{0}$ as 1.

**Table S3.** The combinations of hyper parameters and conditions examined for DOT image reconstruction using the VB algorithm. We controlled i) the covariance matrix mode, ii) the modeling error weight, iii) the radius of brain activity (smoothing radius), and iv) whether updating observation noise variance or not. We also tested the results using different MN method’s solutions as the initial values.

| **VB** | **00** | **10** | **11** | **12** | **13** | **14** | **15** | **16** |
| --- | --- | --- | --- | --- | --- | --- | --- | --- |
| **Covariance matrix mode** | A | B | B | B | B | C | B | B |
| **Modeling error weight** | 0.002 | 0.002 | 0.002 | 0 | 0 | 0 | 0.002 | 0.002 |
| **Smoothing radius [mm]** | 4 | 4 | 0.1 | 2 | 2 | 2 | 8 | 2 |
| **Update observation noise variance** | Yes | Yes | Yes | Yes | No | Yes | Yes | Yes |

In the pilot study, we set the task period as [5, 15] s, and the permutation test was repeated 1000 times to define the threshold of a significant t-value. We had not removed the motion artifacts (Umeyama and Yamada, 2013) orthogonal to the hemoglobin extinction vectors in the pilot study.

Figure S4 shows the results of reliability evaluation based on Fleiss’s kappa, Gwet’s AC1 coefficient and Dice coefficient from different DOT methods. We found that the MN methods using single-trial data and full noise covariance matrix are more reliable than those using trial-averaged data and/or diagonal noise covariance matrix. The choice of smoothing radius did not have much influence on reliability in our data. Introducing modeling errors in the DOT process is also critical for reliable image reconstruction when using the VB algorithm. In the main experiments, we expanded the modeling error as the regularization term of noise covariance and explored the effect of its weight in detail.


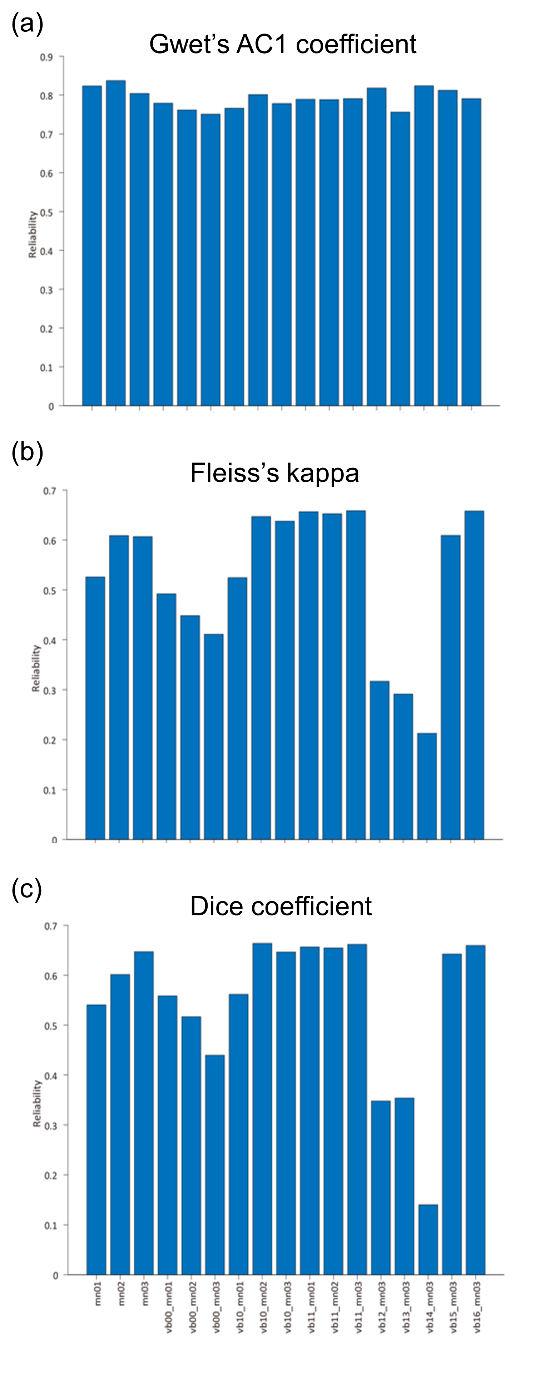


**Figure S4.** Reliability measures for different DOT methods. X-axis labels indicate the DOT methods used. (a) Mean Fleiss’s kappa, (b) mean Gwet’s AC1, (c) mean Dice coefficient. Mean values were calculated across the two monkeys and Hb species (HbO and HbR).

**Supplemental figures of main experiments**


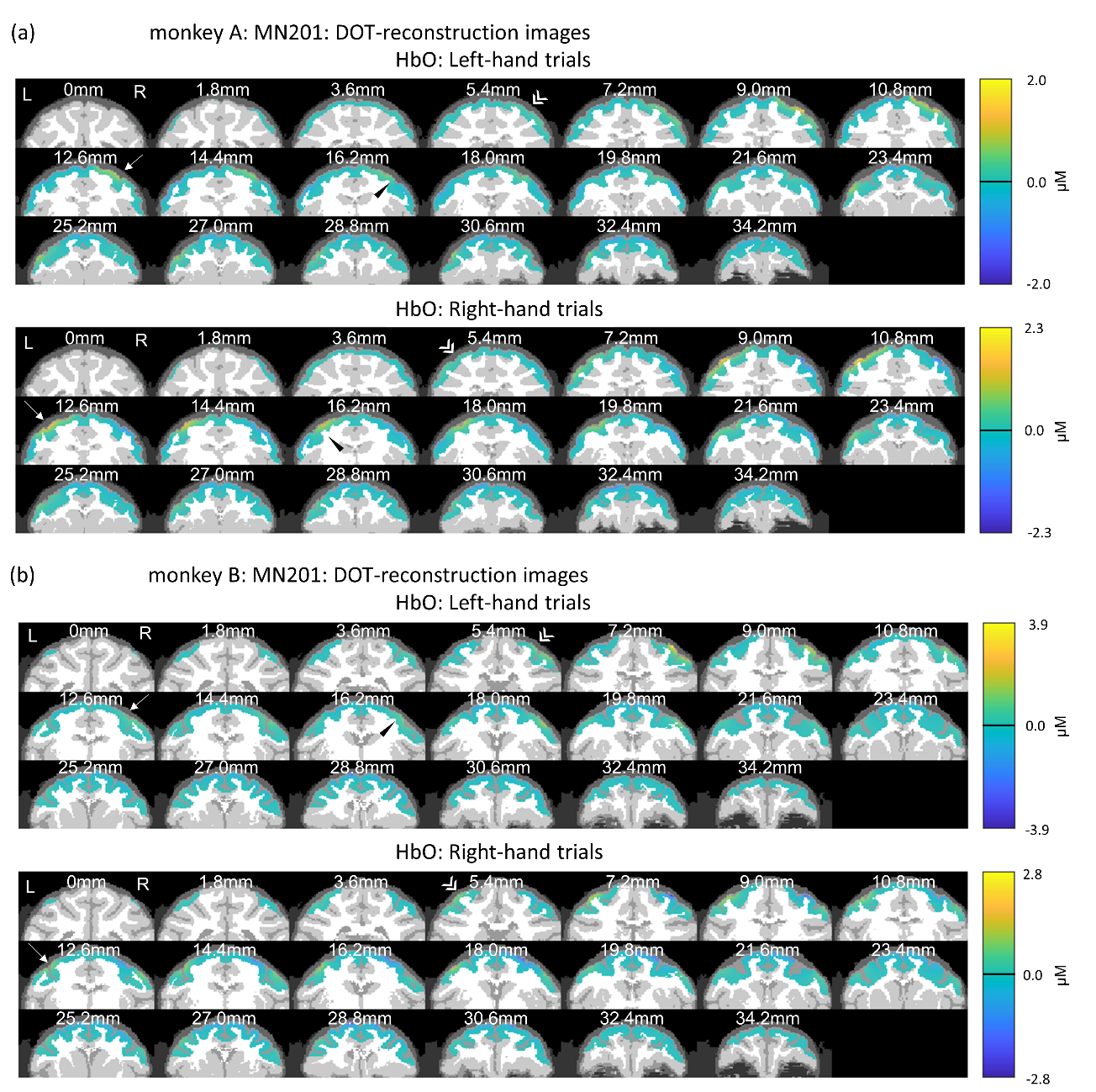


**Figure S5.** Color maps of the original DOT-reconstruction images for HbO using the MN201 method. (a) The mean DOT images for monkey A. (b) The mean DOT images for monkey B. Rows in (a) and (b) represent the mean DOT images averaged from 0 to 10 s. Upper panels depict the left-hand trials and lower panels depict the right-hand trials. Figure configuration is the same as Figure 2 in the main text.


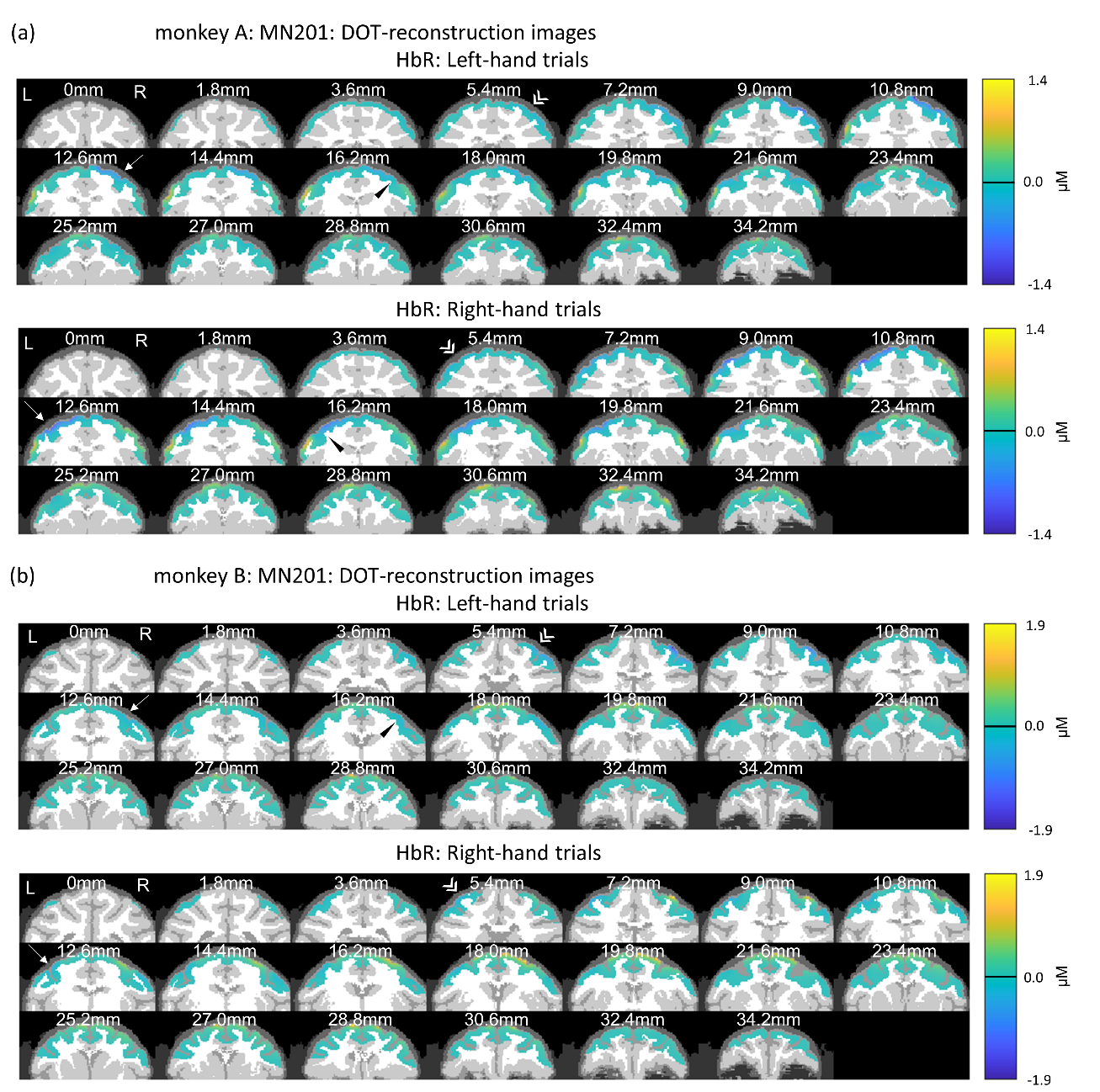


**Figure S6.** Color map of the original DOT-reconstruction images for HbR using the MN201 method. Figure configuration is the same as Figure S5.


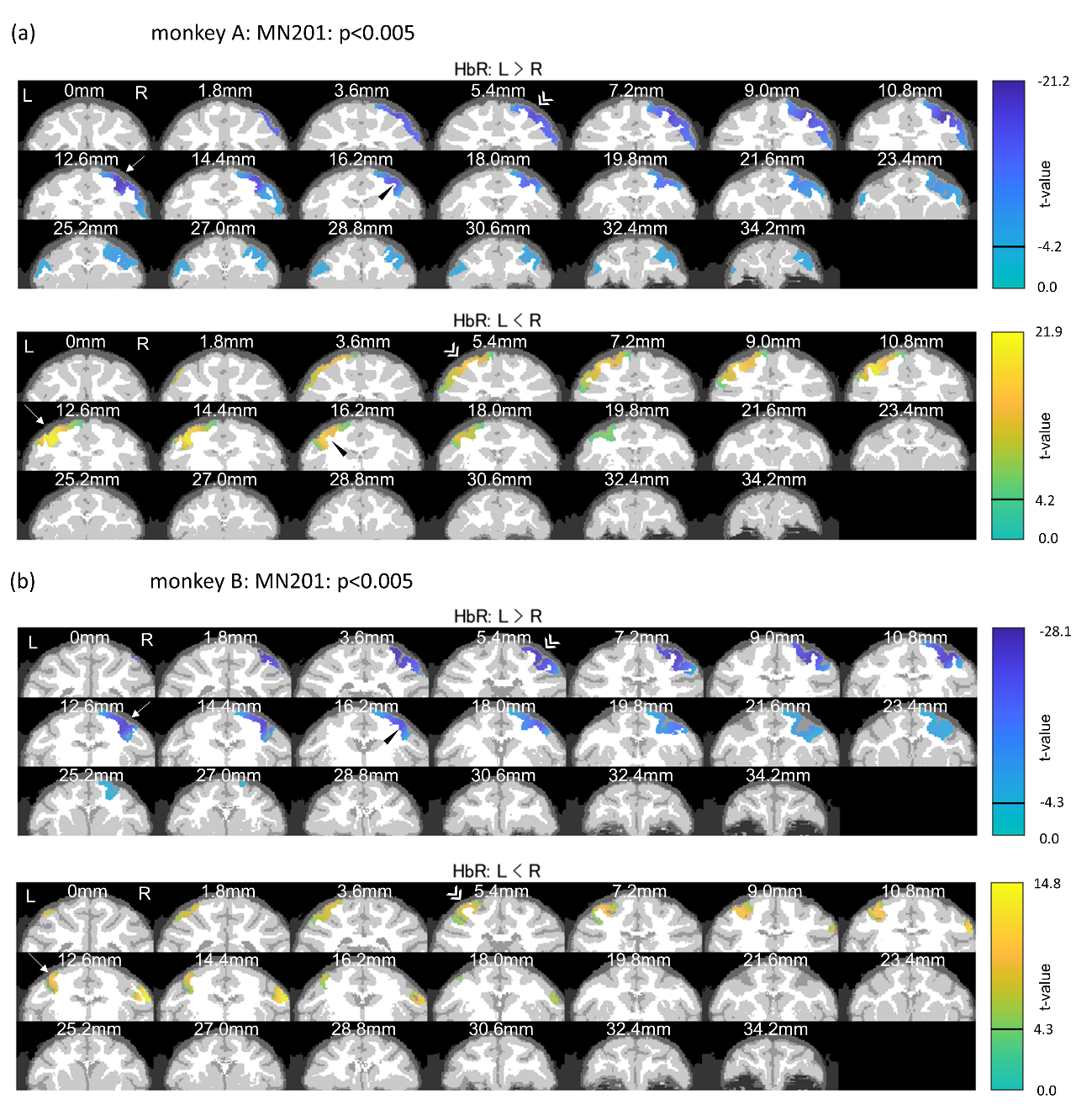


**Figure S7.** Color map of t-values for HbR that exceeded the significant level of 0.5%. Figure configuration is the same as Figure 2 in the main text.

Figure S8 depicts the histogram of t-values images for HbO. The 6 MN methods and 12 VB methods whose parameter of prior confidence was set as 0 are shown in this Figure. The results of MN201 and the VB methods using the solution of MN201 as the initial values (VB201 and VB205) show similar distributions irrespective of the hyper-parameters dedicated to the VB algorithm. The bumps around the both-sides tail are related to the core areas activated by either left-hand or right-hand movement. Results also demonstrate that the t-value distributions are asymmetric for monkey B; absolute t-values are lower for voxels showing larger activation in right-hand trials than in left-hand trials. We observed that monkey B could pick up a food pellet through a narrow slit more smoothly by the right-hand than by the left-hand. Whenever the animal used its left-hand, it hit its hand against the slit’s wall several times. Monkey B also frequently grabbed the edge of the chair with its left-hand while reaching the food with its right-hand. The imbalance between task difficulty and/or hand use could explain the asymmetry of the histogram of t-values for monkey B.


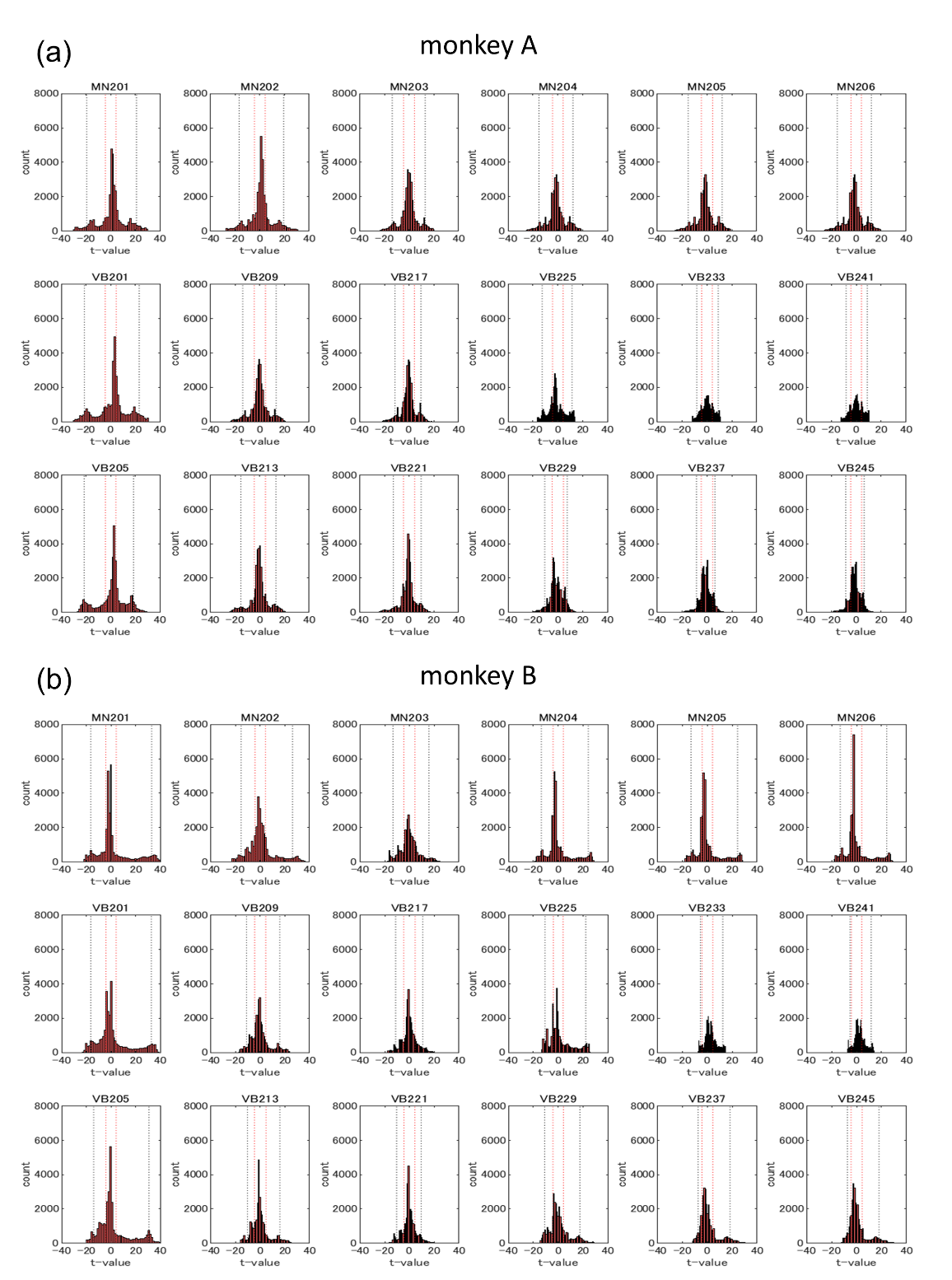


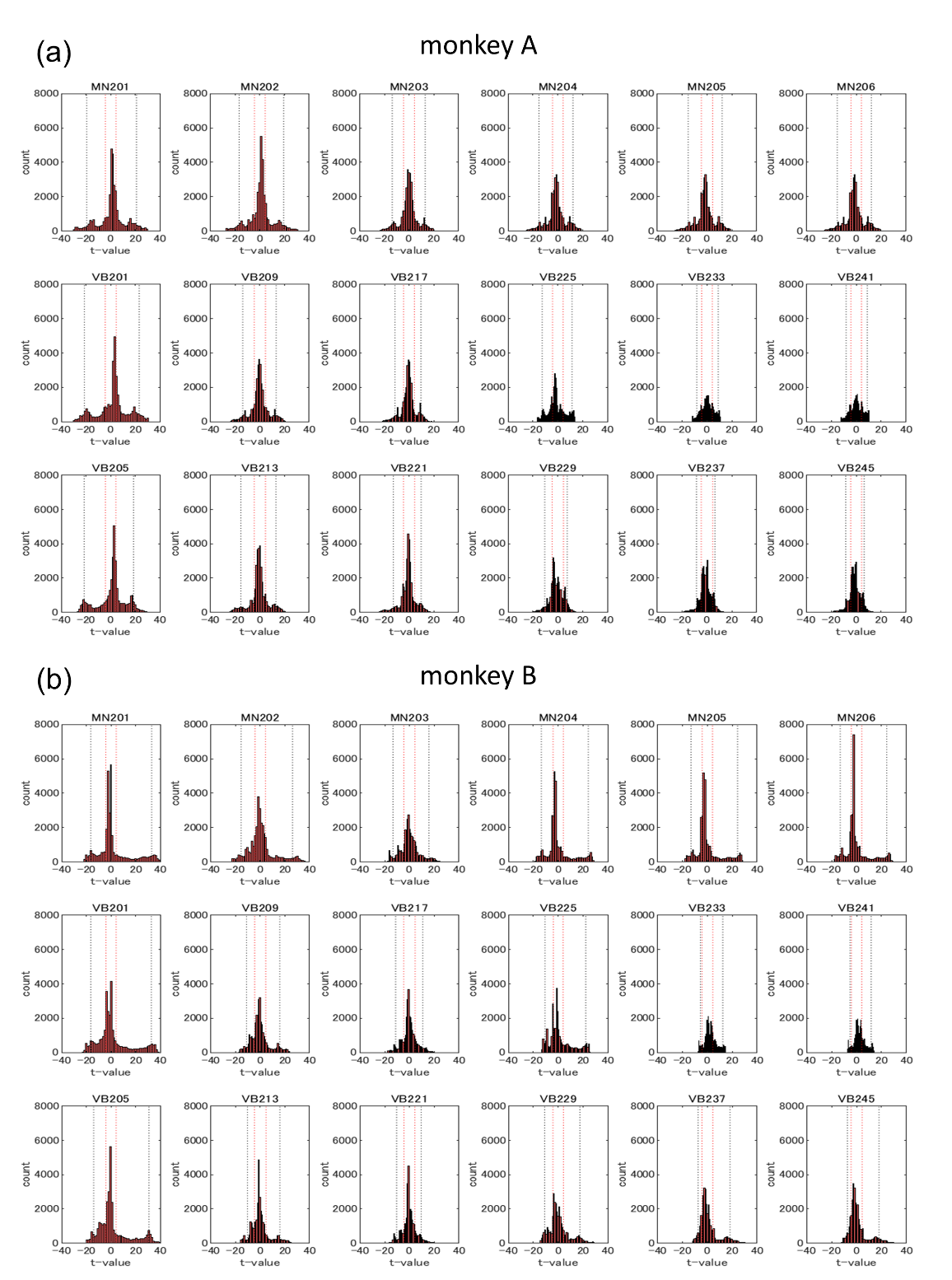


**Figure S8.** Histograms of t-value images for monkey A (a) and monkey B (b). The results (HbO) from different DOT methods (6 MN methods and 12 VB methods) are shown in this figure. Positive scores indicate that HbO changes in left-hand trials were larger than those in right-hand trials, and negative scores indicate vice versa. The red dotted lines indicate the threshold at a significance level of 0.5 %. The black dotted lines indicate the border of the voxels whose t-values are the top 5% highest and lowest values.


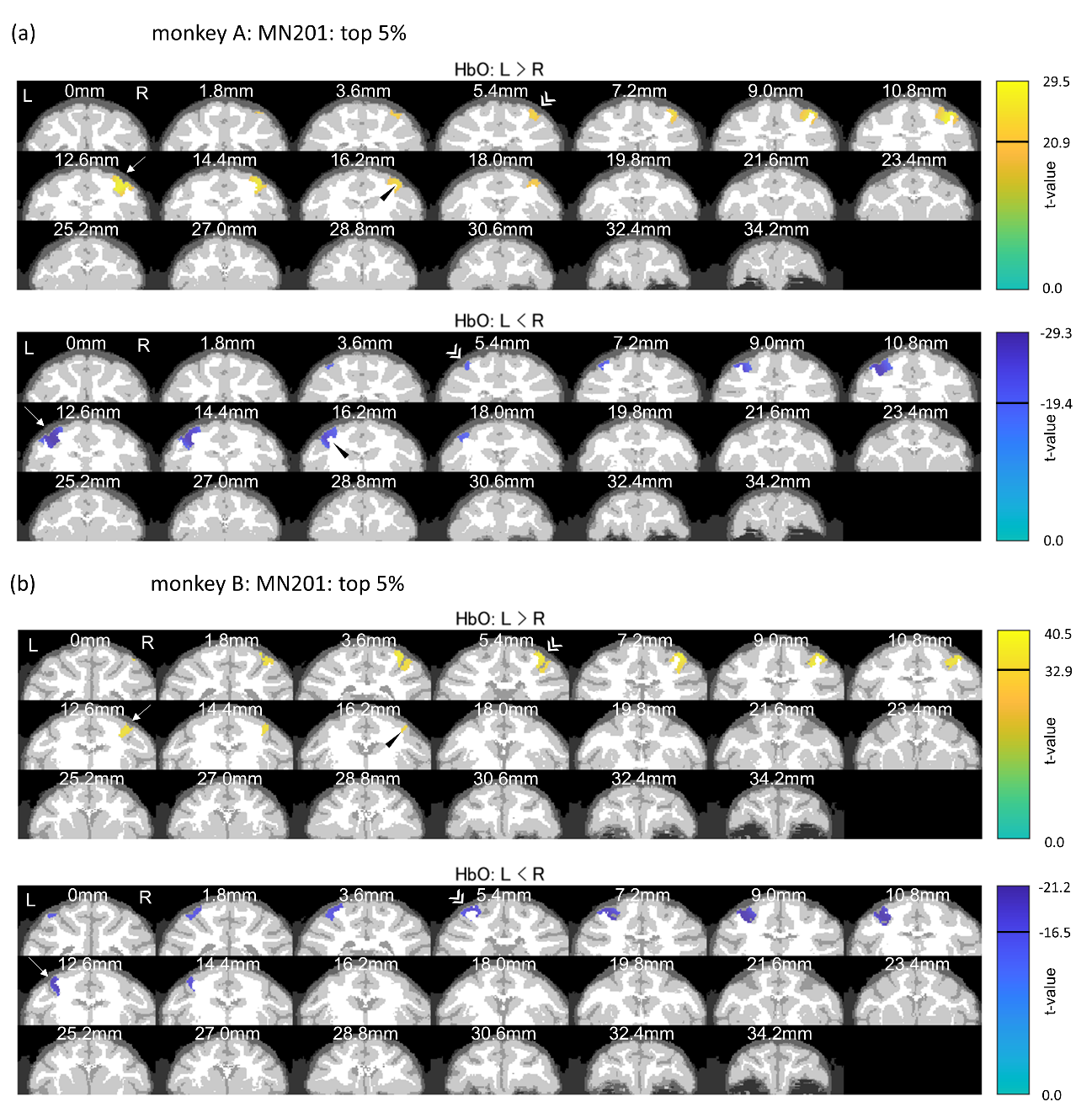


**Figure S9.** Color maps of core activated areas for HbO. The core activated areas were defined by voxels with the top 5% highest and lowest t-values. Figure configuration is the same as Figure 2 in the main text.


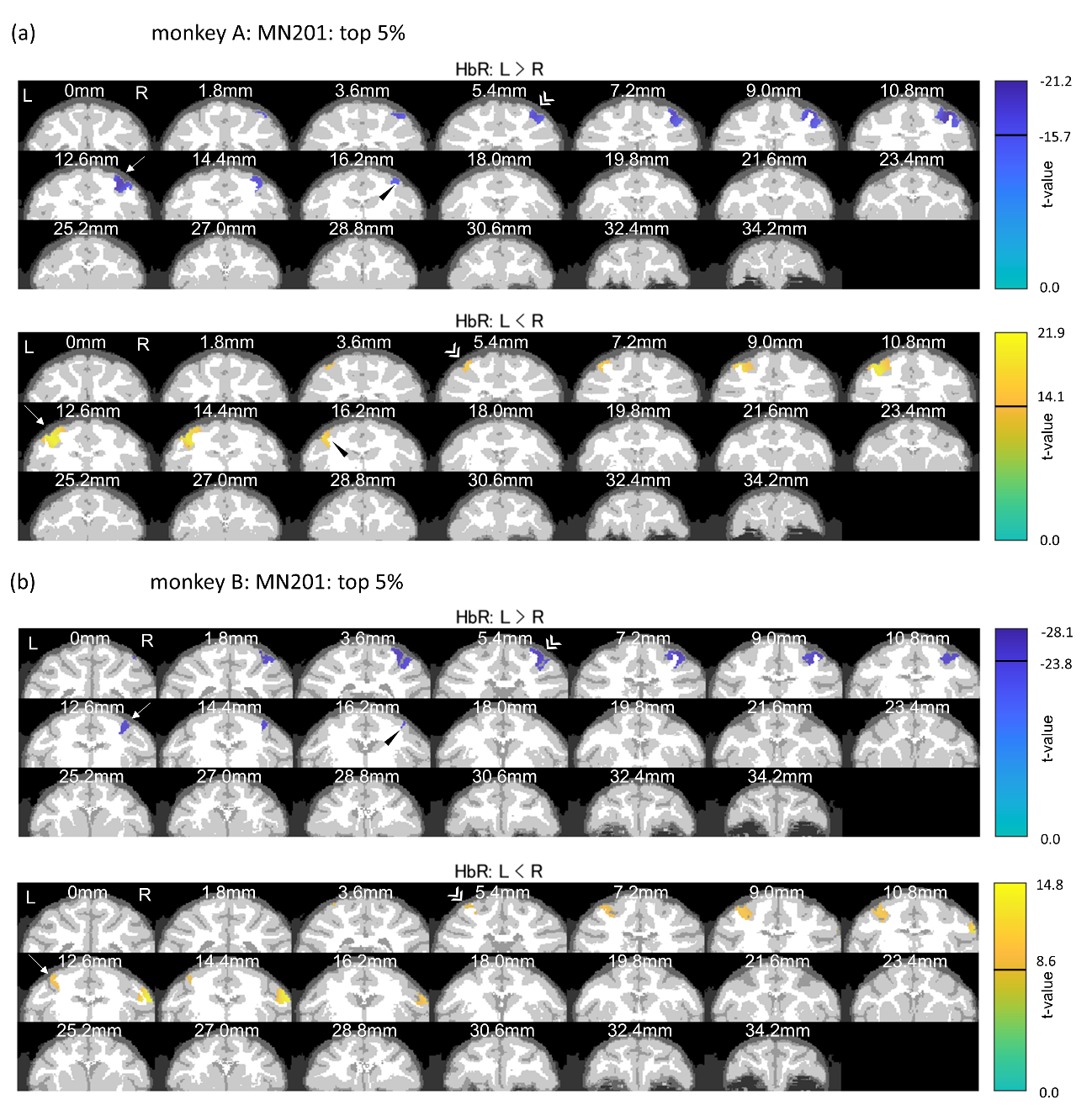


**Figure S10.** Color maps of core activated areas for HbR. Figure configuration is the same as Figure 2 in the main text.

**Table S4.** Statistical tables of three-way ANOVA conducted on the results of the VB methods. X1 = initial value, X2 = smoothing radius, X3 = prior confidence.

| Correlation coefficient (HbO and HbR) | F | P value | Overlap rate (HbO and HbR) | F | P value |
| --- | --- | --- | --- | --- | --- |
| X1 | 20.19779289 | 5.73E-12 | X1 | 55.66575477 | 3.27E-33 |
| X2 | 8.955163856 | 0.003947559 | X2 | 2.991903984 | 0.085622319 |
| X3 | 0.05366976 | 0.98346666 | X3 | 0.704300755 | 0.550788224 |
| X1*X2 | 3.94462546 | 0.003559745 | X1*X2 | 3.714151322 | 0.003308047 |
| X1*X3 | 0.029908329 | 0.999999998 | X1*X3 | 0.525222758 | 0.923792249 |
| X2*X3 | 0.053591854 | 0.983501447 | X2*X3 | 0.704300755 | 0.550788224 |
| Gwet’s AC1 | F | P value | Fleiss’s kappa | F | P value |
| X1 | 15.54975158 | 1.92E-12 | X1 | 36.41831495 | 9.62E-25 |
| X2 | 2.19848284 | 0.140126 | X2 | 0.027939396 | 0.867463776 |
| X3 | 0.365675135 | 0.777874347 | X3 | 0.059484513 | 0.980919481 |
| X1*X2 | 0.563786413 | 0.72764165 | X1*X2 | 0.638610155 | 0.670554161 |
| X1*X3 | 0.221590752 | 0.999099221 | X1*X3 | 0.058410258 | 0.999999871 |
| X2*X3 | 0.511832299 | 0.674693743 | X2*X3 | 0.133502751 | 0.939982292 |
| Dice coefficient | F | P value | Detection rate | F | P value |
| X1 | 42.43064914 | 1.29E-27 | X1 | 65.5090057 | 7.38E-37 |
| X2 | 0.8952834 | 0.345485286 | X2 | 0.107693746 | 0.743216963 |
| X3 | 0.06140069 | 0.980024862 | X3 | 0.172491554 | 0.91486309 |
| X1*X2 | 0.766112188 | 0.575515413 | X1*X2 | 1.161191846 | 0.330699288 |
| X1*X3 | 0.014921211 | 1 | X1*X3 | 0.104821482 | 0.99999255 |
| X2*X3 | 0.1029752 | 0.95821418 | X2*X3 | 0.370460237 | 0.774422662 |
| ICC (1,1)  (ROI-based analysis on t-value images) | F | P value | I2C2  (voxel-based analysis on original DOT images) | F | P value |
| X1 | 32.46012569 | 1.03E-22 | X1 | 26.2346079 | 2.88E-19 |
| X2 | 0.162126567 | 0.687746974 | X2 | 3.715759909 | 0.055683581 |
| X3 | 0.153480551 | 0.927325201 | X3 | 0.222372587 | 0.880748088 |
| X1*X2 | 1.571659222 | 0.17103453 | X1*X2 | 5.227716385 | 0.000179903 |
| X1*X3 | 0.120936596 | 0.999980576 | X1*X3 | 0.645460351 | 0.83349082 |
| X2*X3 | 0.153496345 | 0.927315005 | X2*X3 | 0.124307131 | 0.945629784 |

**Table S5.** Statistical tables of Dennett’s test between MN201 and the VB methods (p value).

|  | **VB201** | **VB202** | **VB203** | **VB204** | **VB205** | **VB206** | **VB207** | **VB208** |
| --- | --- | --- | --- | --- | --- | --- | --- | --- |
| Gwet’s AC1 | 0.477 | 0.422 | 0.360 | 0.356 | 0.108 | 0.0719 | 0.193 | 0.417 |
| Fleiss’s kappa | 0.977 | 0.970 | 0.957 | 0.941 | 0.657 | 0.560 | 0.804 | 0.942 |
| Dice coefficient | 1.00 | 1.00 | 1.00 | 1.00 | 0.996 | 0.994 | 1.00 | 1.00 |
| Detection rate | 0.0715 | 0.0549 | 0.0453 | 0.0578 | 0.0523 | 0.0427 | 0.0656 | 0.102 |

**
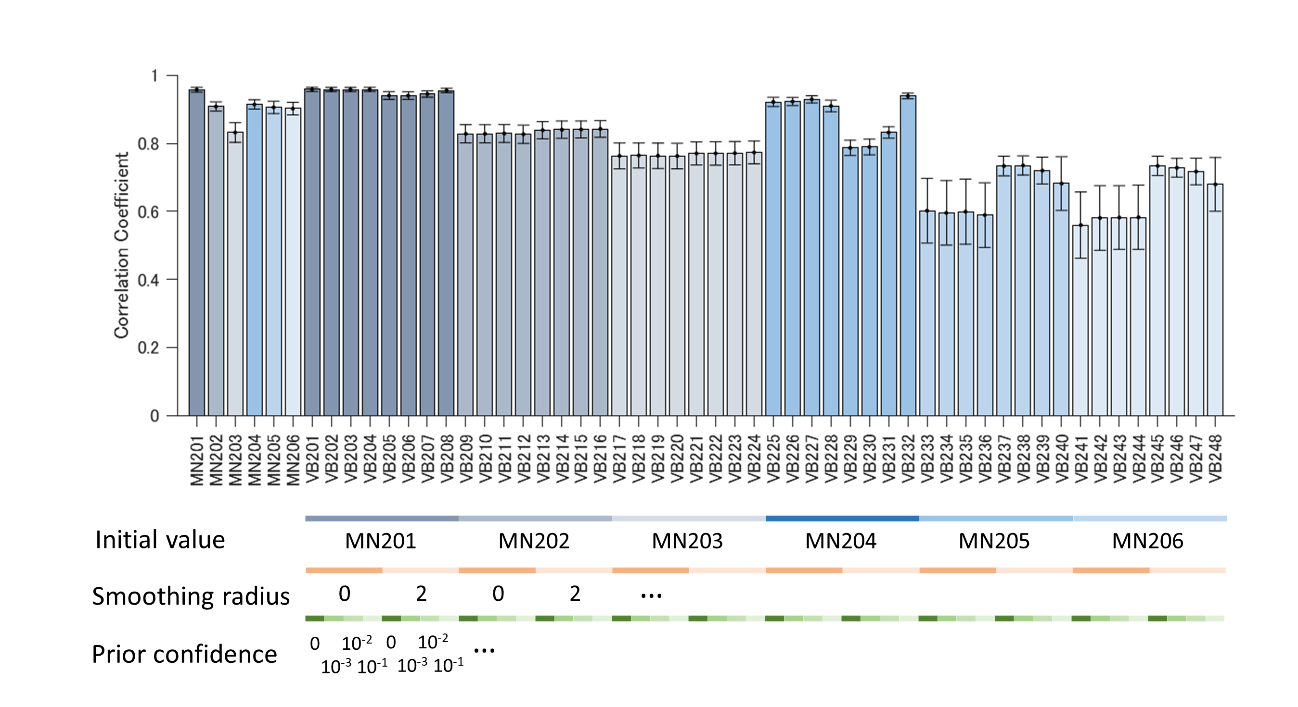
**

**Figure S11.** ROI-based analysis of reliability evaluation. The mean correlation coefficient of the hand modulation intensity between different recording days are shown as graphs. The intensity measure of hand modulation is defined as the minimum and maximum t-values within each brain area. Mean values were calculated across the two animals and Hb species. Error bars indicate the standard error.

**
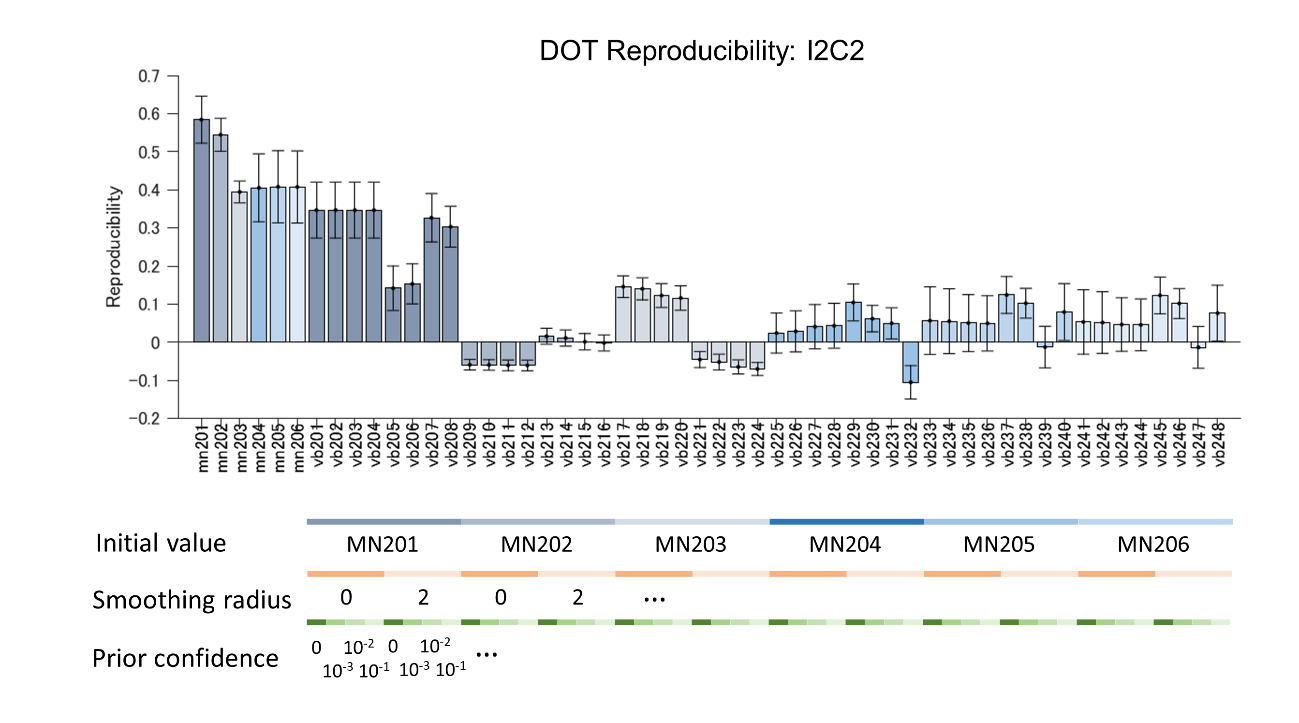
**

**Figure S12.** Intraclass correlation coefficients between the mean DOT images from left-hand and right-hand trials are shown as bar graphs. Mean values were calculated across the two animals and Hb species. Error bars indicate the standard error.


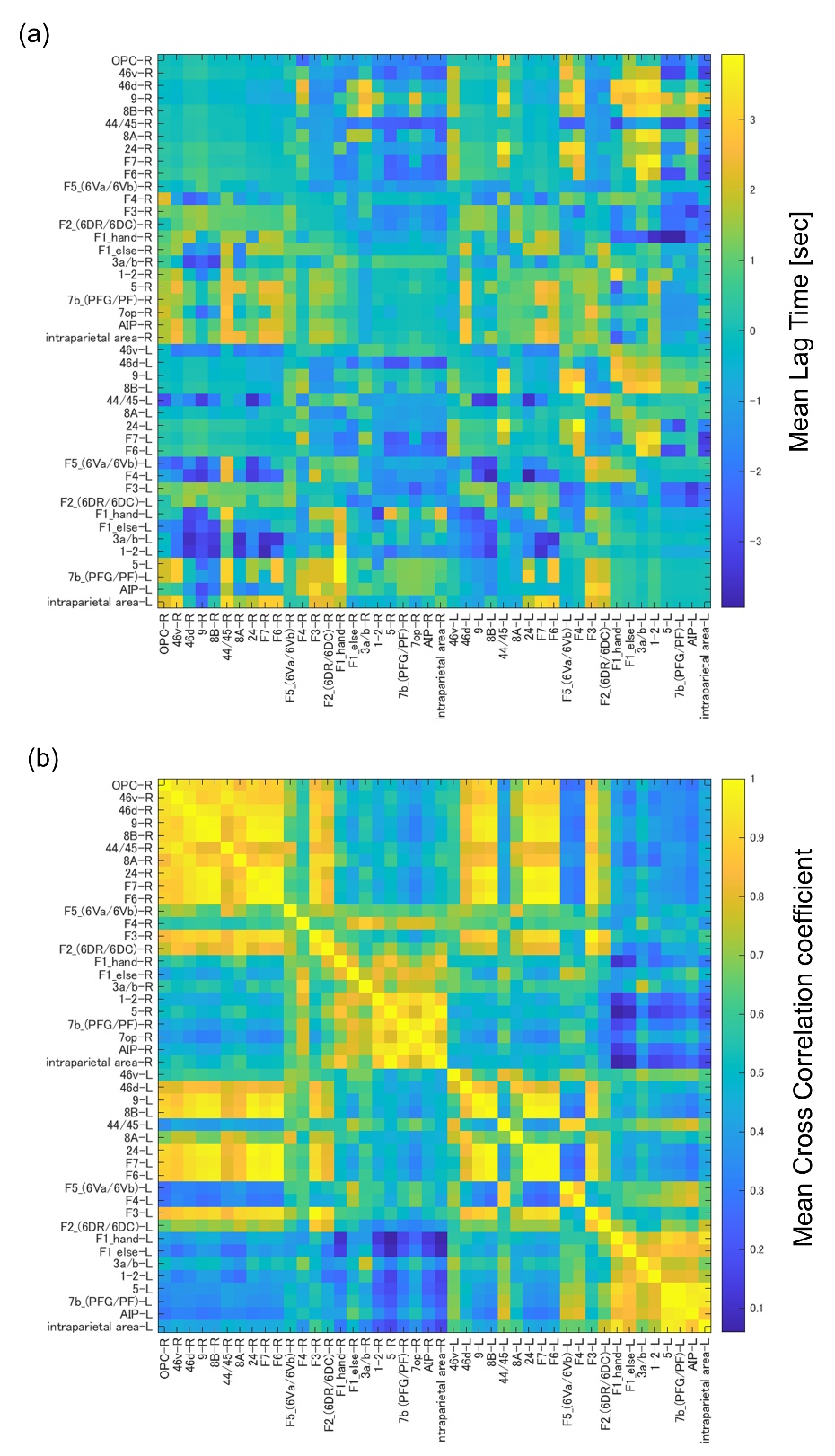


**Figure S13.** Results of cross-correlation analysis based on time series data of 44 brain areas. (a) Mean lag time between different areas. Lag time was determined as the time that provides the maximum cross correlation between the time series of HbO/HbR changes in two areas. The hot color indicates that the HbO/HbR changes in areas in the y-axis are delayed compared with those in the areas in the x-axis, and the cold color indicates vice versa. (b) Mean maximum cross-correlation coefficient between different areas. Mean values were calculated across the two animals, Hb species, and tasks.


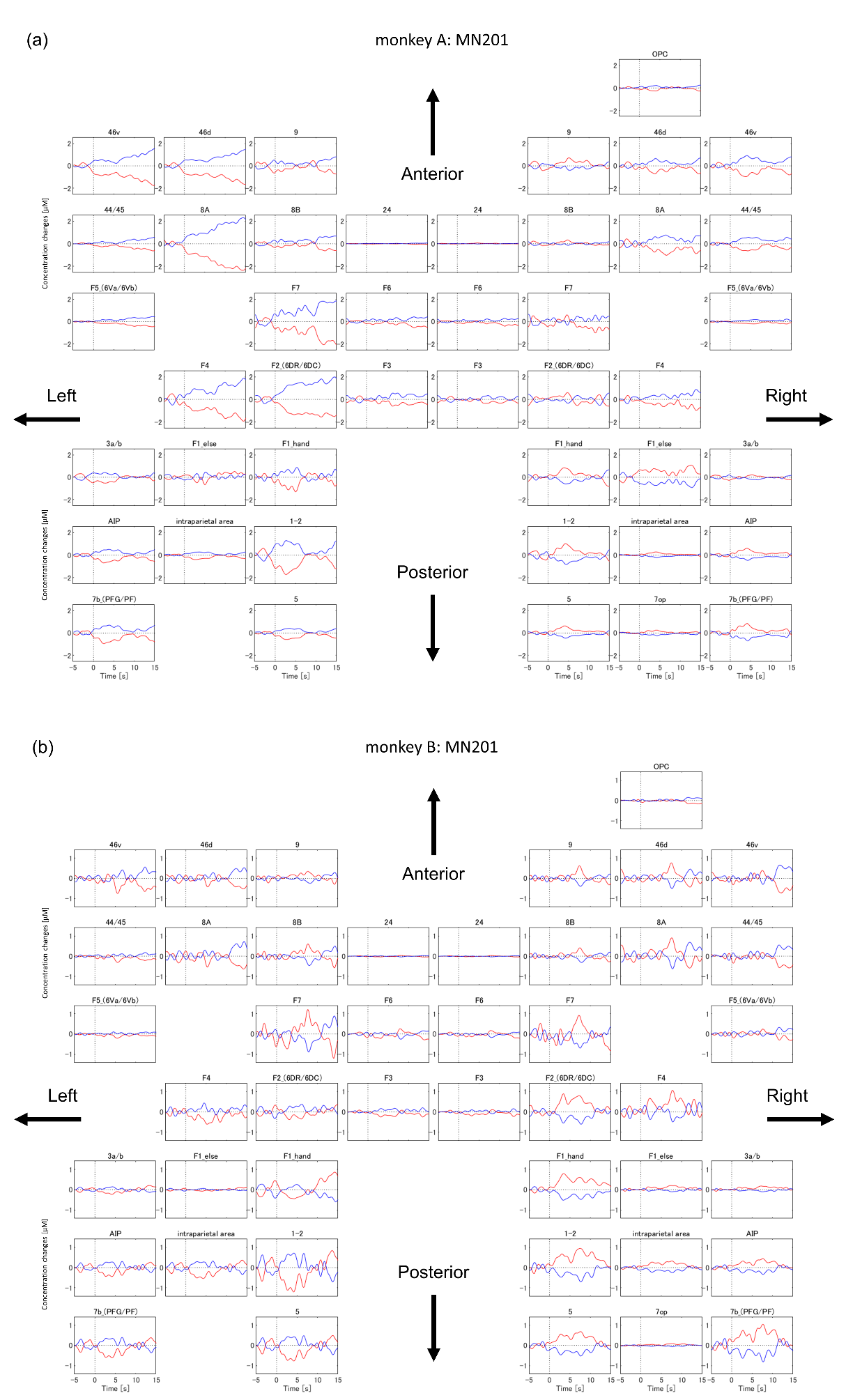


**Figure S14.** Single-trial time plot of HbO/HbR changes in each brain area. (a) Time plots for monkey A. (b) Time plots for monkey B.
